# Pistachio genomes provide insights into nut tree domestication and ZW sex chromosome evolution

**DOI:** 10.1016/j.xplc.2022.100497

**Published:** 2022-11-26

**Authors:** Salih Kafkas, Xiaokai Ma, Xingtan Zhang, Hayat Topçu, Rafael Navajas-Pérez, Ching Man Wai, Haibao Tang, Xuming Xu, Mortaza Khodaeiaminjan, Murat Güney, Aibibula Paizila, Harun Karcı, Xiaodan Zhang, Jing Lin, Han Lin, Roberto de la Herrán, Carmelo Ruiz Rejón, Jerson Alexander García-Zea, Francisca Robles, Coral del Val Muñoz, Agnes Hotz-Wagenblatt, Xiangjia Jack Min, Hakan Özkan, Elmira Ziya Motalebipour, Hatice Gozel, Nergiz Çoban, Nesibe Ebru Kafkas, Andrej Kilian, HuaXing Huang, Xuanrui Lv, Kunpeng Liu, Qilin Hu, Ewelina Jacygrad, William Palmer, Richard Michelmore, Ray Ming

**Affiliations:** 1 Department of Horticulture, Faculty of Agriculture, University of Çukurova, Adana 01330, Turkey; 2 Center for Genomics and Biotechnology, Haixia Institute of Science and Technology, School of Future Technology, Fujian Agriculture and Forestry University, Fuzhou, China; 3 Key Laboratory of Orchid Conservation and Utilization of National Forestry and Grassland Administration, Fujian Agriculture and Forestry University, Fuzhou, China; 4 Departamento de Genética, Facultad de Ciencias, Campus de Fuentenueva s/n, 18071 Granada, Spain; 5 Department of Plant Biology, University of Illinois at Urbana-Champaign, Urbana, IL 61801, USA; 6 Key Laboratory of the Ministry of Education for Coastal and Wetland Ecosystems, College of the Environment and Ecology, Xiamen University, Xiamen 361102, China; 7 Department of Computer Science, University of Granada, Granada, Spain; 8 Andalusian Research Institute in Data Science and Computational Intelligence (DaSCI Institute), 18014 Granada, Spain; 9 German Cancer Research Center, Omics IT and Data Management Core Facility, Heidelberg, Germany; 10 Department of Biological Sciences, Youngstown State University, Youngstown, OH 44555, USA; 11 Department of Field Crops, Faculty of Agriculture, University of Çukurova, Adana 01330, Turkey; 12 Pistachio Research Institute, Şahinbey, Gaziantep 27060, Turkey; 13 Diversity Arrays Technology, University of Canberra, Canberra, ACT, Australia; 14 Genome Center, University of California Davis, 451 Health Sciences Drive, Davis, CA 95616, USA

**Keywords:** *Pistacia vera*, pistachio, sequencing, reference genome, sex chromosome, domestication

## Abstract

Pistachio is a nut crop domesticated in the Fertile Crescent and a dioecious species with ZW sex chromosomes. We sequenced the genomes of *Pistacia vera* cultivar (cv.) Siirt, the female parent, and *P. vera* cv. Bagyolu, the male parent. Two chromosome-level reference genomes of pistachio were generated, and Z and W chromosomes were assembled. The ZW chromosomes originated from an autosome following the first inversion, which occurred approximately 8.18 Mya. Three inversion events in the W chromosome led to the formation of a 12.7-Mb (22.8% of the W chromosome) non-recombining region. These W-specific sequences contain several genes of interest that may have played a pivotal role in sex determination and contributed to the initiation and evolution of a ZW sex chromosome system in pistachio. The W-specific genes, including *defA*, *defA-like*, *DYT1*, two *PTEN1*, and two tandem duplications of six *VPS13A* paralogs, are strong candidates for sex determination or differentiation. Demographic history analysis of resequenced genomes suggest that cultivated pistachio underwent severe domestication bottlenecks approximately 7640 years ago, dating the domestication event close to the archeological record of pistachio domestication in Iran. We identified 390, 211, and 290 potential selective sweeps in 3 cultivar subgroups that underlie agronomic traits such as nut development and quality, grafting success, flowering time shift, and drought tolerance. These findings have improved our understanding of the genomic basis of sex determination/differentiation and horticulturally important traits and will accelerate the improvement of pistachio cultivars and rootstocks.

## Teaser

This study reports the generation of chromosome-level reference genomes of the female *Pistacia vera* cultivar Siirt and the male *P. vera* cultivar Bagyolu and the assembly of Z and W chromosomes. W-specific genes such as *defA*, *defA-like*, and *DYT1* were detected, and selective sweeps that underlie agronomic traits such as nut development and quality, grafting success, flowering time shift, and drought tolerance were identified.

## Introduction

The genus *Pistacia* (Anacardiaceae) consists of 11 species (Zohary, 1952; Parfitt and Badenes, 1997; Kafkas and Perl-Treves, 2001; Kafkas, 2006, 2019), of which *P. vera* is the most economically important owing to its edible nuts. Pistachio is often known as the “King of Nuts” (Contenson, 1983). *P. vera* is a dioecious species with a haploid chromosome number of n = 15 (Basr Ila et al., 2003) and a genome size of 1C = 660 Mbp (Horjales Luaces et al., 2003). The value of the pistachio nut has reached approximately $10 billion USD annually (FAOSTAT, 2022; http://faostat.fao.org/). Currently, the United States, Iran, Turkey, and Syria produce more than 90% of the world pistachio crop. Despite its long history of cultivation and economic importance, little is known about the domestication and sex determination system of pistachio. Our current knowledge of pistachio domestication is largely derived from a population genomic analysis (Zeng et al., 2019) and archaeological evidence (Kashaninejad and Tabil, 2011).

The availability of a high-quality genome sequence and germplasm resources for pistachio will enable the detection of candidate genes for domestication that are related to important agronomic traits. Previous studies have demonstrated that *P. vera* possesses the ZW sex chromosome system (Kafkas et al., 2015; Khodaeiaminjan et al., 2017) with heteropicnotic sex chromosome pairs (Sola-Campoy et al., 2015). Pistachio could serve as a valuable model species for sex determination because the ZW system is very rare in dioecious plant species (Ming et al., 2011).

In the last decade, studies on sex determination in plants have been reported in 20 families and a total of 48 species (Ming et al., 2011; Harkess and Leebens-Mack 2017; Muyle et al., 2017). To date, among 28 species with heteromorphic sex chromosomes, there are only a few species with ZW chromosomes, including pistachio. Thus, it is very significant to discover dioecious plant species, particularly crops such as pistachio, with ZW sex determination systems and to explore their evolution, domestication, population genetics, and beneficial mutations related to important agronomic traits at the genome level (Natri et al., 2019). Also, such work will greatly assist breeding programs, nursery management, and germplasm collection, especially in the identification and completion of sex chromosome evolution in the genus *Pistacia*, and in establishing tools and genomic resources for determining the sex of seedlings at an early stage in *Pistacia* breeding programs.

Here, we describe the high-quality genome assembly and annotation of *P. vera* cv. Siirt, a female cultivar widely planted in Turkey, and *P. vera* cv. Bagyolu, a male cultivar, and we characterize the sex determination region (SDR) in pistachio. High-density linkage maps from three segregating populations enabled characterization of the SDRs in female ZW and male ZZ genomes. Resequencing of *P. vera* accessions and other *Pistacia* species provided details on the origin and breeding history of pistachio as well as genomic and genetic changes resulting from human domestication. The additional insights presented here about the function of the pistachio SDR and ZW system will improve our understanding of SDRs and ZW sex chromosome evolution.

## Results

### Genome sequencing and assembly

**Figure 1:**
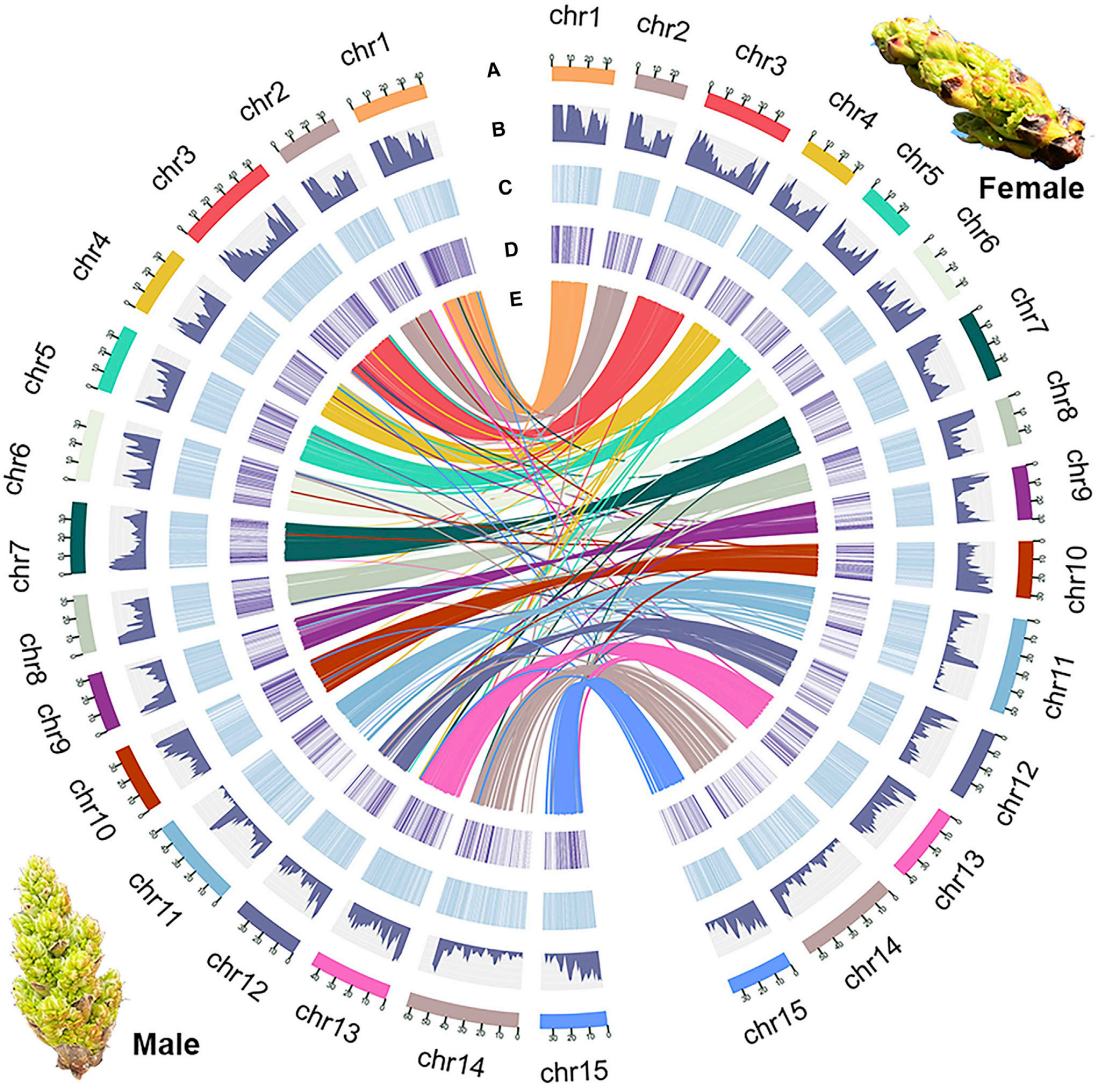
Chromosomal features of the genomes of pistachio cultivars Siirt (right) and Bagyolu (left). **(A)** Chromosomes. **(B)** Gene densities. **(C)** Transposable elements. **(D)** Gene expression. **(E)** Syntenic regions between the genomes of pistachio cultivars Siirt and Bagyolu. The inner lines show syntenic blocks in homologous chromosomes between the genomes of pistachio cultivars Siirt and Bagyolu.

The genomes of *P. vera* cv. Siirt (female) and cv. Bagyolu (male) were sequenced at 90× and 76× coverage, respectively, using the PacBio RS II platform. We initially assembled the two genomes using CANU (Supplemental Tables 1 and 2) and an algorithm that improves the contiguity of heterozygous genomes. This resulted in an assembly size for the Siirt genome of 614.1 Mb with a contig N50 of 679.5 kb and an assembly size for the Bagyolu genome of 622.4 Mb with a contig N50 of 92.1 kb. We further extended the Siirt contigs using a variety of mate pair libraries with different insert sizes, yielding 1787 scaffolds with an N50 of 1.51 Mb. The chromosome-level genome assemblies were achieved using high-throughput chromosome capture (Hi-C) technology, which increased the assembly of cv. Bagyolu to a scaffold N50 of 39.8 Mb with 99.9% (623.3/623.4 Mb) of sequences anchored onto 15 pseudo-chromosomes (Table 1; Figure 1). The assembly of cv. Siirt was further improved using a reference-guided assembly strategy based on the male chromosome-level assembly, resulting in a scaffold N50 of 38.7 Mb with 99.3% (592.6/597.0 Mb) of sequences anchored onto 15 pseudo-chromosomes. Validation of these genome assemblies using six individual high-density genetic linkage maps as well as a consensus map from three segregating F1 populations (Supplemental Table 3) revealed that 96.2% (10 795/11 222) of the single-nucleotide polymorphism (SNP) markers were aligned onto the Siirt Hi-C assembly and 90.5% (10 111/11 169) were aligned onto the Bagyolu assembly (Supplemental Figures 1 and 2), indicating high consistency between the genetic maps and Hi-C scaffolding results.

**Table 1:** Assembly and annotation statistics for *Pistacia vera* cultivars Siirt and Bagyolu.

	Siirt (female)	Bagyolu (male)
**Assembly**		
Number of scaffolds	50	28
Longest scaffold	57 779 128	62 820 281
Scaffold N50 (Mb)	38.7	39.8
Assembly length (Mb)	596.0	623.4
% of sequences anchored onto pseudochromosomes	99.3	99.9
**Annotation**		
Predicted gene models	29 695	29 996
Average gene length (bp)	3580	3427
Average CDS length (bp)	212.5	203.83
Average exon number per gene	6.03	6.51
Average exon length (bp)	243.3	248.4
Average intron length (bp)	420.3	328.37
Alternative splicing genes	7882	6263

Repetitive elements	Total length and percentage	Total length and percentage
Retrotransposons	90 138 480 (14.86%)	97 653 449 (16.01%)
DNA transposons	5 165 372 (0.86%)	10 228 705 (1.72%)
Integrated virus	2 411 868 (0.4%)	1 741 570 (0.31%)
Simple repeats	14 143 278 (2.30%)	13 504 649 (2.17%)
Unknown	123 983 109 (20.44%)	127 164 923 (20.40%)
Main satellite DNA families	46 343 068 (7.64%)	50 396 105 (8.05%)
Total	282 185 175 (46.5%)	300 689 401 (48.66%)

Non-coding RNAs	Copies	Copies
rRNAs	544	245
tRNAs	600	797
miRNAs	963	977
snRNAs	128	124
snoRNAs	2263	2330

Both assemblies represented gene space well. We performed CEGMA (Parra et al., 2007) analysis and found 228 (91.9%) complete gene models from the core set of 248 ultra-conserved eukaryotic genes in both genomes (Supplemental Table 4). In addition, 1253 (91.1%) and 1274 (92.7%) of 1375 conserved BUSCO (Simão et al., 2015) genes were present in the assemblies of cvs. Siirt and Bagyolu, respectively (Supplemental Table 5).

### Gene prediction and annotation

By performing two rounds of MAKER (Cantarel et al., 2008), 29 695 and 29 996 gene models were obtained for the chromosome-level assemblies of cvs. Siirt and Bagyolu, respectively (Supplemental Table 6). Average gene model lengths for cvs. Siirt and Bagyolu were 3580 and 3427 bp with 6.03 and 6.51 exons per gene (Table 1). BUSCO analysis revealed 93.9% and 92.5% completeness of the Siirt and Bagyolu annotations (Supplemental Table 5). In addition, small non-coding RNAs (Table 1, Supplemental Tables 7A–7I, 8A–8D, and 9A–9D), repetitive elements (Supplemental Tables 10, 11A, and 11B), putative splicing variants (Supplemental Tables 12, 13A, 13B, 14, 15A, and 15B), and nucleotide-binding site (NBS)-encoding resistance genes (Supplemental Tables 16, 17, 18, 19A, and 19B) were annotated in the two genomes.

### Defining the sex chromosome and SDR

The correlation curves between physical and genetic distances showed clear suppression of recombination along chromosome 14 in the female Siirt genome for each of the six genetic maps (Supplemental Figure 3). The other chromosomes exhibited no such severe recombination suppression, indicating that Chr14 is a pistachio ZW sex chromosome.

**Figure 2:**
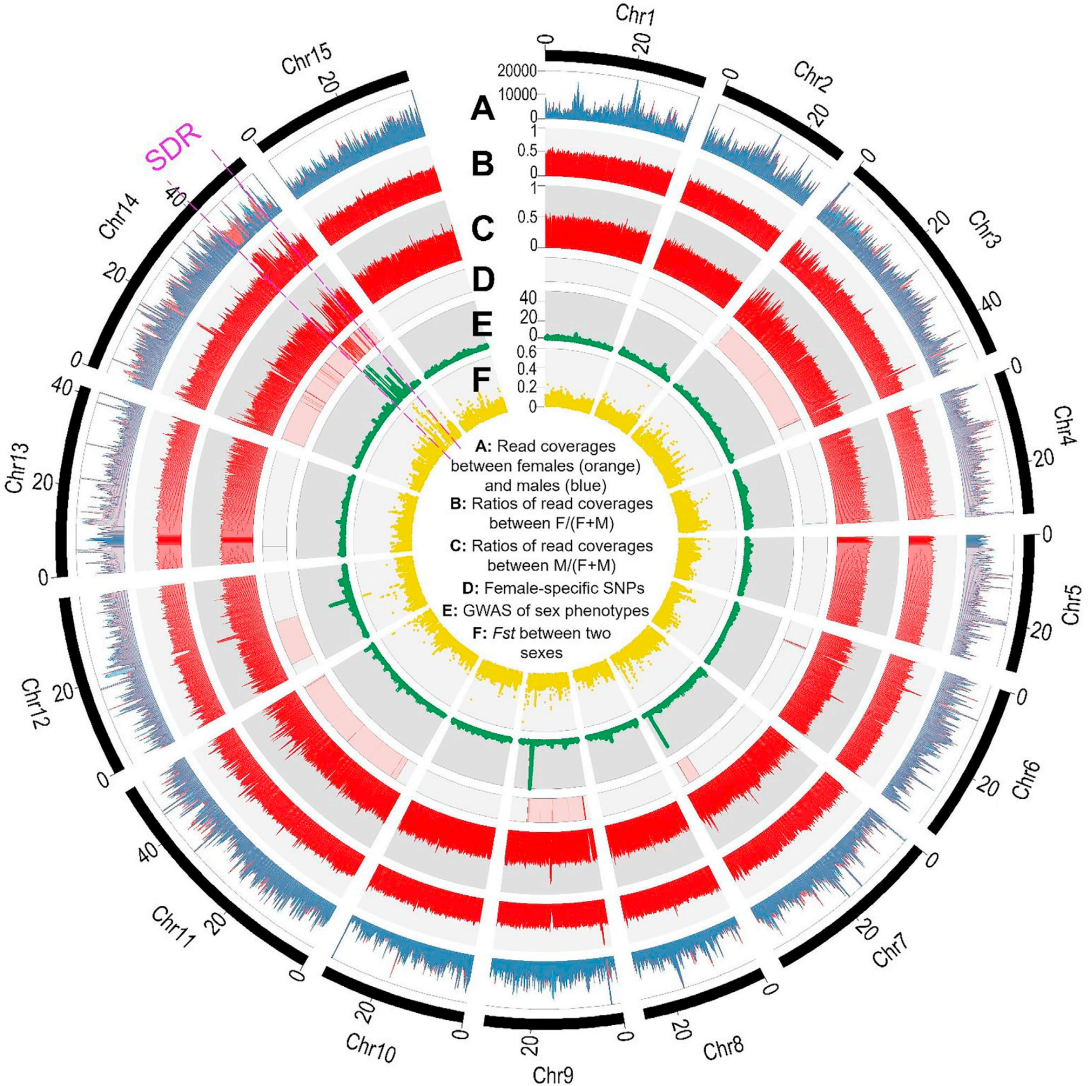
Definition of sex chromosomes (Chr14) and the SDR on the female pistachio Siirt genome. From outer to inner ring of the circos plot. **(A)** Read coverages of pooled females (orange) and males (blue). **(B)** Ratios of read coverage F/(F + M) between females and males. **(C)** Ratios of read coverage M/(F + M) between males and females. **(D)** Female-specific SNPs. **(E)** Plot of −log10(P) values of a genome-wide association study (GWAS) between the two sex phenotypes (including 22 resequenced females and 22 resequenced males). **(F)***Fst* between female and male sex phenotypes. The results indicate that Chr14 is the pistachio sex chromosome. The putative SDR (Chr14: 39.98–52.68 Mb; size = 12.70 Mb) is defined by the above lines of evidence along Chr14 of the female pistachio Siirt genome.

**Figure 3:**
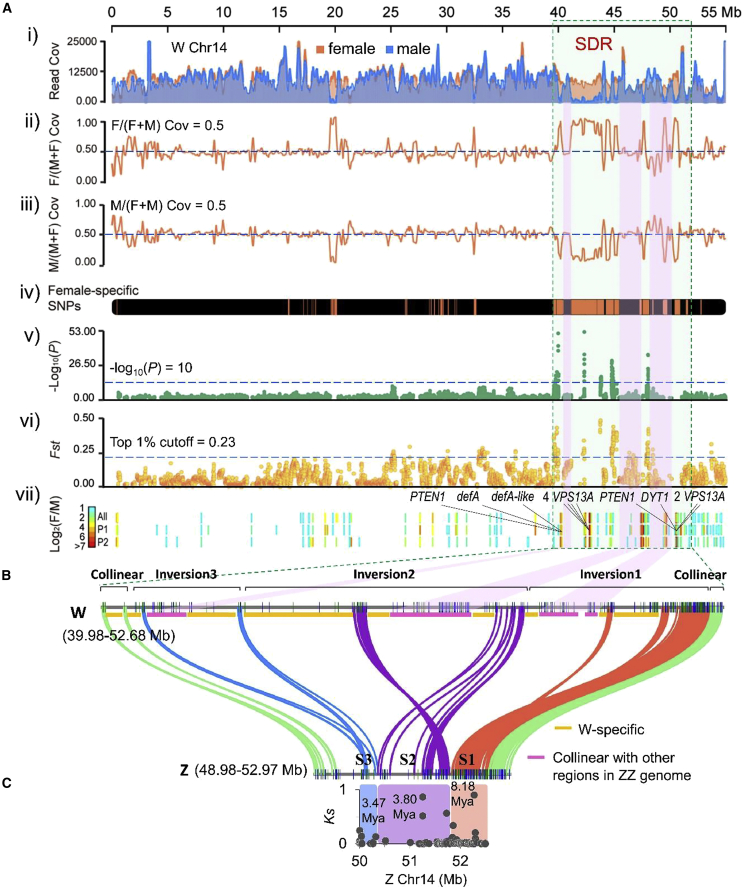
Genomic features of the SDR and its Z counterpart. **(A)** Evidence and boundaries of the SDR along Chr14 of the female pistachio Siirt genome. From top to bottom of figure: i) read coverage of pooled resequenced females (nine individuals) and males (nine individuals); ii) the ratio F/(F + M) of female and male read coverage, with a cutoff threshold of 0.5; iii) the ratio M/(F + M) of male and female read coverage, with a cutoff threshold of 0.5; iv) female-specific SNPs showing continuous blocks; v) GWAS plot between the two sex phenotypes, with cutoff of −log10(*P*) = 10; vi) *Fst* between females and males, with the top 1% cutoff peak; vii) DEGs (differentially expressed genes, showing log_2_F/M > 0) between females and males in two different flower developmental periods (P1, P2) and throughout all periods (All). Highlighted genes are W-specific SDR genes, including *defA* (pistachio.v30109300), *defA-like* (pistachio.v30109290), *DYT1* (pistachio.v30112090), two *PTEN1* genes (pistachio.v30112050A and pistachio.v30109260), and two tandem duplications of *VPS13A* (two paralogs: pistachio.v30112100 and v30112110; and four paralogs: pistachio.v30109730, v30109740, v30109750, and v30109760). **(B)** Collinear genomic landscape of the SDR (39.98–52.68 Mb) and its Z counterpart (48.98–52.97 Mb). Microsynteny between gene pairs of W chromosome sequences compared with their counterparts on the Z chromosome (192 W–Z gene pairs). Three large chromosomal inversions on W Chr14: 40647874-52416681 (size = 11.77 Mb) and corresponding Z Chr14: 50002496-52514087 (size = 2.51 Mb). W-specific sequences are marked with yellow blocks, and regions collinear with other regions except Z-counterpart regions of the ZZ genome are marked with pink blocks. **(C)** Plots of synonymous site divergence (*Ks*) of paired genes on the physical map of gene order along the Z chromosome. Inversions and collinear regions of the pistachio sex chromosomes Z and W with different *Ks* values and divergence times (Mya) are shown. The plots show three evolutionary strata: strata 1, 2, and 3 (S1, 8.18 Mya; S2, 3.80 Mya; S3, 3.47 Mya).

To further define the SDR, the read coverages between pooled resequenced females (nine individuals of F1 population) and males (nine individuals of F1 population) were plotted. The ratios of read coverage between the two sexes (cutoff threshold F/(F + M) = 0.5, while M/(F + M) = 0.5) showed a continuous block of read coverage divergence, with both F/(F + M) and M/(F + M) ratios deviating from 0.5 between the sexes at Chr14: 39.98–52.68 Mb (12.70 Mb) on the Z/W chromosome, revealing the SDR region (Figures 2 and 3A i, ii, and iii; Supplemental Figures 4 and 5). In the 12.70-Mb SDR, some disjunct regions showed male read reduction/absence (F/(F + M) > 0.5; M/(F + M) < 0.5) compared with genome-wide read coverage ratios (cutoff threshold F/(F + M) = 0.5, while M/(F + M) = 0.5), representing the W-specific region. Some disjunct regions showed higher read coverage ((F/(F + M) < 0.5; M/(F + M) > 0.5)) or equal read coverage (F/(F + M) ≈ 0.5; M/(F + M) ≈ 0.5) in males compared with females, representing the W homologs of Z sequences. Nine W-specific sequences were clearly defined (Figure 3A and 3B; Supplemental Figure 6; Supplemental Table 20A).

In the SDR region, a high −log_10_(*P*) score (cutoff of −log_10_(*P*) = 10) of a genome-wide association study (GWAS) of 2 sex phenotypes (22 resequenced females and 22 resequenced males) showed clear peaks. The top 1% peak (cutoff = 0.23) of genetic differentiation (*Fst*) between them also showed clear peaks at the same region. This region also included the continuous blocks of female-specific SNPs (Figures 2 and 3A; Supplemental Figure 7A). Chr14 was therefore defined as a pistachio ZW sex chromosome, and the two boundaries of the SDR were defined at Chr14: 39.98–52.68 Mb (12.70 Mb) according to the cutoff threshold F/(F + M) = 0.5 and M/(F + M) = 0.5. The regions of signals in the GWAS and *Fst* analyses are smaller than this defined region, likely owing to missing genotypes in W-specific regions such as Chr14: 50.43–51.35 Mb. Nonetheless, these disjunct regions had continuous blocks of female-specific SNPs and read coverage divergence, which supported the SDR definition. The strong female-biased/specific expression at this region also supported this definition (Figure 3A vii; Supplemental Figure 5; Supplemental Table 20A).

To verify whether the SDR was assembled chimerically, we calculated the sequence identity between the SDR and its Z counterpart by blastN searching with a 1-kb sliding window. The average sequence identity for each contig on the SDR was calculated by averaging the identity value of each window. The overall sequence identity between the SDR and its Z counterpart was only 70.64% (Supplemental Figure 6A). Three small contigs (Chr14: 40723001–40758658; Chr14: 51450189–51506788; and Chr14: 51506889–51528470) with average sequence identity >95% were located in collinear regions that were part of the pseudo-autosomal region and expected to be homologous. The lack of contiguous homologous sequences between the SDR and its Z counterpart indicated that the SDR was unlikely to be a chimeric assembly.

In addition to the SDR, there were other regions (such as Chr14: 19.65–20.57 Mb; Figures 2 and 3A; Supplemental Figures 4, 5, and 7) with read coverage divergence in which both F/(F + M) and M/(F + M) ratios deviated from 0.5 between the two sexes, and some other regions had high −log_10_(*P*) scores of GWAS signals (such as Chr7: 36.00–36.08 Mb and Chr9: 23.35–23.70 Mb; Figure 2). However, these regions showed no evidence of multiple signals supporting them as the SDR (Figure 2; Supplemental Figures 4, 5, and 7).

The SDR boundaries were also confirmed by comparative collinearity analyses between the W-SDR in Chr14 of cultivar “Siirt” and its Z counterpart of cultivar “Bagyolu” using MCScanX (Wang et al., 2012a); the SDR was at Chr14: 39979216–52678755 (12.70 Mb), whereas the Z counterpart was at Chr14: 48975748–52967355 (3.99 Mb) (Figure 3B; Supplemental Figures 6B and 7B). Microsynteny analysis between W and Z gene models revealed three inversions in the SDR at Chr14: 40647874–52416681 (11.77 Mb, 387 genes): inversion 1 at Chr14: 49032998–52416681 (3.38 Mb, 170 genes), inversion 2 at Chr14: 42858892–48612698 (5.75 Mb, 173 genes), and inversion 3 at Chr14: 40647874–42816399 (2.17 Mb, 44 genes). Their corresponding counterparts in the Z chromosome were at Chr14: 50002496–52514087 (2.51 Mb, 184 genes): inversion 1 counterpart at Chr14: 51779783–52514087 (0.73 Mb, 102 genes), inversion 2 counterpart at Chr14: 50320242–51728478 (1.41 Mb, 66 genes), and inversion 3 counterpart at Chr14: 50002496–50266641 (0.26 Mb, 16 genes). In addition, 2 collinear regions were identified in the W Chromosome, Chr14: 39979216–40460393 (0.48 Mb) and Chr14: 52417733–52678755 (0.26 Mb); their corresponding regions in the Z chromosome were Chr14: 48975748–49411952 (0.44 Mb) and Chr14: 52537909–52967355 (0.43 Mb) (Figure 3B; Supplemental Figure 7B).

To confirm the correct assembly of the inversions, we estimated the coverage of PacBio reads mapping onto the junction regions of these structural variations. For each 5-kb window, the junctions of inversions 1 and 2 and of inversions 2 and 3 were covered by an average of 100.71 reads and 138.67 reads, respectively; the junctions of inversion 1 and the collinear region and of inversion 3 and the collinear region were covered by an average of 85.5 and 71.97 reads, respectively (Supplemental Table 20B), validating the correct assembly of these inversions.

Combining the collinear relationship between the SDR and the Z counterpart, as well as read coverage divergence between the female and male genomes, nine W-specific regions and four regions collinear with other regions in the ZZ genome except the Z counterpart were clearly defined and have determined the current gene order in the SDR (Figure 3A and 3B; Supplemental Figure 7A and 7B; Supplemental Table 20A). Four regions collinear with other regions, including Chr14: 41009767–41566853 (0.56 Mb), Chr14: 45933462–47505147 (1.57 Mb), Chr14: 49048829–49640049 (0.59 Mb), and Chr14: 49958154–50081910 (0.12 Mb), may be potential translocations from either Chr14 or Chr6 autosome regions (Figure 3B; Supplemental Figure 7B; Supplemental Table 20A).

The divergence time between the SDR and its counterpart was estimated using W and Z paired genes across the SDR by calculating synonymous substitution rates (*Ks*) and applying the mutation rate mu = 7.5 × 10^−10^ per site per year for the genus *Pistacia* (Parfitt and Badenes 1997). Our analysis revealed that the median divergence times of inversions 1, 2, and 3 were 8.18, 3.80, and 3.47 Mya, respectively, forming three evolutionary strata (strata 1, 2, and 3) (Figure 3C; Supplemental Figure 7C).

### Gene content of W-specific sequences and candidates for sex determination/differentiation

We annotated 427 protein-coding genes in the SDR, including 191 Z and W paired genes and 65 W-specific genes in the SDR, whereas there were 78 Z-specific genes in the Z counterpart (Supplemental Table 20A). The average gene density was 27.46 ± 14.82 genes/Mb in the W SDR, which was much lower than the 66 ± 42.59 genes/Mb in the Z counterpart and the genome-wide average of 33.71 ± 33.63 genes/Mb in the female Siirt genome and 47.77 ± 30.97 genes/Mb in the male Bagyolu genome (all three comparisons are *P* < 0.05, pairwise *t-*test). GO term analysis indicated enrichment of W-specific genes (*P* < 0.05, Fisher’s exact test) for “phospholipid dephosphorylation,” “vernalization response,” “regulation of flower development,” and “regulation of reproductive process” in the Biological Process domain (Supplemental Figure 8A; Supplemental Table 21A); for “trans-Golgi network” and “organelle subcompartment” in the Cellular Component domain (Supplemental Figure 8B; Supplemental Table 21B); and for “phosphatidic acid binding,” “phospholipid binding,” and “tetraketide alpha-pyrone synthase activity” in the Molecular Function domain (Supplemental Figure 8C; Supplemental Table 21C).

We propose that the W-specific genes represented by enriched GO terms related to reproductive processes and related functions may have contributed to the evolution of dioecy in pistachio. For example, the pistachio.v30112090 gene encodes a putative bHLH transcription factor; mutation of its *Arabidopsis* homolog *DYT1* (*DYSFUNCTIONAL TAPETUM1*) results in a male sterile mutant with abnormal anther morphology beginning at anther development stage 4 (Zhang et al., 2006). Two genes (pistachio.v30112050 and pistachio.v30109260) are homologous to the tumor suppressor homolog *AtPTEN1*, which encodes a Tyr phosphatase that is essential for pollen development in *Arabidopsis*; suppression of its expression by RNA interference led to pollen cell death after mitosis (Gupta et al., 2002). Two tandem duplications involved two paralogs (pistachio.v30112100 and v30112110) and four paralogs (pistachio.v30109730, v30109740, v30109750, and v30109760) of *VPS13A*, whose ortholog *VPS52* showed male gametophytic mutants characterized by very short pollen tubes (Lobstein et al., 2004; Guermonprez et al., 2008). By checking mapping read coverage of resequenced accessions in regions of *DYT1*, two *VPS13A*, and two *PTEN1*, we found that these reads were present in female accessions but absent in male accessions (Supplemental Figure 9).

In addition, two W-specific genes with strongly female-specific/biased expression encoding the floral homeotic transcription factor DEFICIENS (*defA*) (pistachio.v30109300) and a floral homeotic *defA*-like protein (pistachio.v30109290) are present in all 44 resequenced female pistachio accessions and absent in all 23 male accessions, except for 2 possibly misidentified accessions (Supplemental Table 22). The same patterns were also verified in female and male accessions of 10 congener species in the same genus (Supplemental Table 22). By analyzing the read mapping coverage of these 2 genes, we found that mapped reads were present in all resequenced female accessions and absent in all resequenced male accessions (Supplemental Figure 9), which we confirmed by PCR (Supplemental Figure 10). Interestingly, one study has shown that mutations in *defA* result in the transformation of stamens into carpels in *Antirrhinum* flowers (Sommer et al., 1990). Thus, we propose the *DYT1*, six *VPS13A*, two *PTEN1*, and *defA* and *defA*-like genes as candidate genes for sex determination/differentiation in pistachio.

Repetitive sequences are abundant in the pistachio SDR, representing 77.46% of the mixed W sequence and 66.24% of the Z sequence (Supplemental Tables 23 and 24), higher than the genome-wide averages of 64.98% for the female Siirt genome and 60.46% for the male Bagyolu genome. However, the collinear region has a much lower fraction of repetitive sequences, with 57.18% in W and 49.89% in its Z counterpart. Conversely, the inverted region has a much higher fraction of repetitive sequences, with 77.57% in W and 60.32% in its Z counterpart. This difference in the abundance of repetitive sequences between the inverted and collinear regions is mainly due to the accumulation of retrotransposons. The LTR/ERV1, LINE/CR1, LINE/L1-Tx1, and LINE/RTE-BovB elements were specifically accumulated in the W inversion region rather than the Z inversion and the collinear regions (Supplemental Tables 23 and 24).

### Population genomics and domestication

#### Origin, dispersal, and breeding history of pistachio

**Figure 4:**
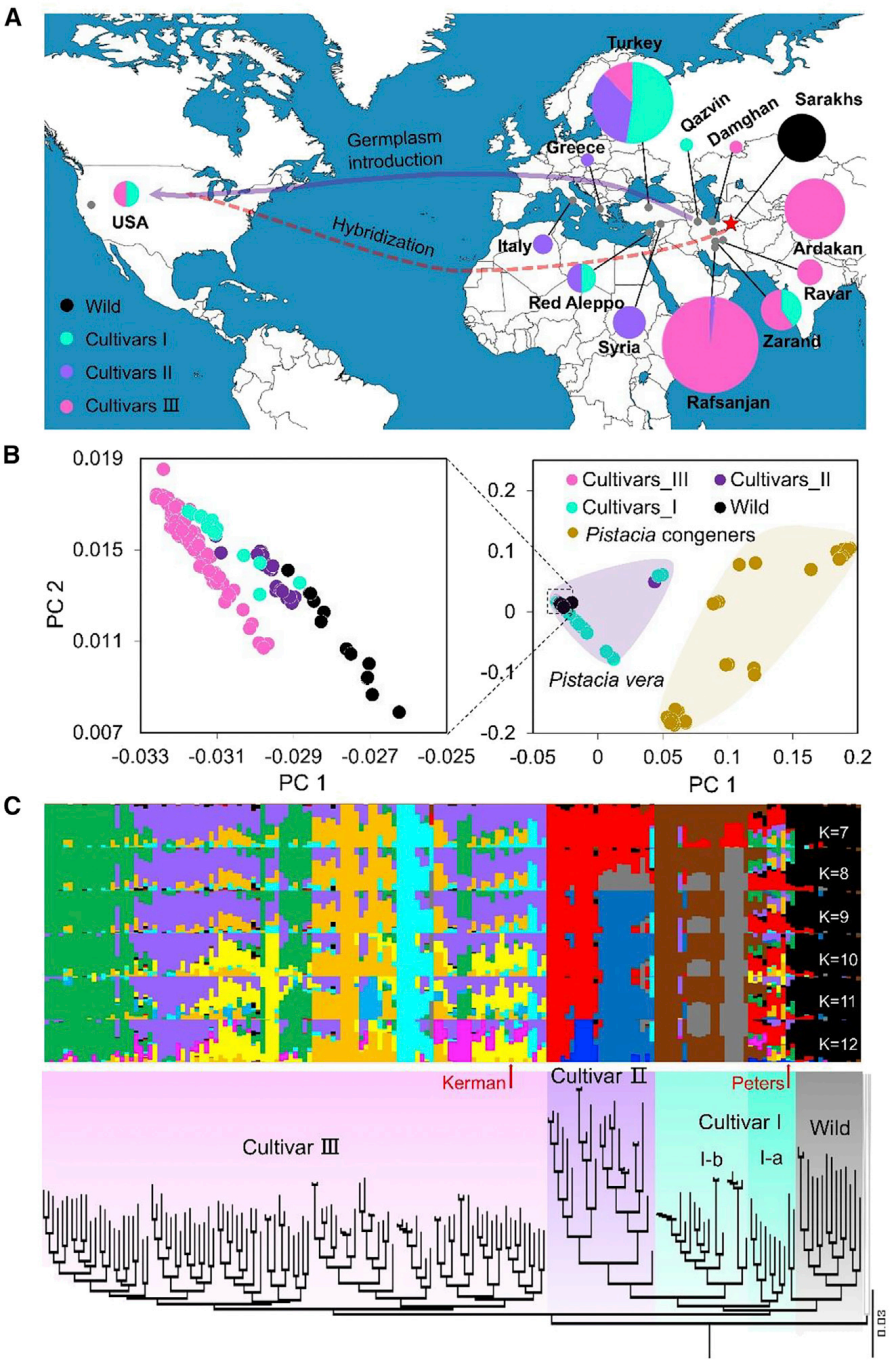
Population genomic analyses of 225 *Pistacia* accessions: 14 wild and 160 domesticated *P. vera* accessions and 51 accessions from 11 closely related *Pistacia* species. **(A)** Proposed origin and domestication route of *P. vera* varieties and the main cultivars in the USA. The diameters of the pie charts are proportional to the number of accessions, up to a maximum of 100 accessions. **(B)** PCA shows clear separation of the *P. vera* population and other sampled *Pistacia* relatives. Wild *P. vera* and its cultivars were also well separated. **(C)** Population structure and phylogenetic relationships of 160 *P. vera* cultivars and 14 wild accessions, with optimal clusters as K = 11. Two clusters belong to subgroup Cultivar_I (which can be divided into Cultivar_Ia and Cultivar_Ib), two clusters belong to Cultivar_II, and six clusters belong to Cultivar_III. Cultivars Peters and Kerman are labeled with red arrows.

We obtained 225 resequenced genomes of diverse *Pistacia* accessions, including 14 wild *P. vera* accessions, 160 domesticated *P. vera* accessions, and 51 accessions from 11 closely related *Pistacia* species (Figure 4A–4C). We identified 5 368 583 high-confidence variants, including 5 059 508 SNPs, 137 195 insertions, and 171 880 deletions. There was an average of 9.17 variants per kb, with 4 536 476 variants (52.25%) in genic regions, including 143 195 synonymous, 156 805 missense, 1996 nonsense, 143 429 silent, and 788 891 intronic variants.

Using *Pistacia* congener species of *P. vera* as the outgroup, we analyzed phylogenetic relationships among 174 *P. vera* accessions (Figure 4C) and found that *P. vera* can be classified into four subgroups: a wild population, Cultivar_I, Cultivar_II, and Cultivar_III. The Cultivar_I group can be further divided into two small subgroups, Cultivar_I-a and Cultivar_I-b, on the basis of phylogenetic and admixture analysis. The phylogenetic tree showed that Cultivar_III, which branches off prior to the wild pistachio accessions, may be an admixture with congener outgroup species. Population admixture analysis showed that the optimal population stratification for the 174 *P. vera* accessions was K = 11 (Figure 4C and Supplemental Figure 11). This clustering was supported by evidence from our principal-component analysis (PCA) (Figure 4B). The PCA clearly separated *P. vera* from other *Pistacia* species and divided *P. vera* cultivars from their wild ancestors. The *Pistacia* congener species clustered like the population of *P. vera* species. Some outlier *P. vera* cultivars that are closer to the congener species may be interspecific hybrids.

The three breeding groups for *P. vera* domestication consist of (1) the Cultivar_I group comprised of 30 accessions including accessions from Zarand in Iran, accessions from Turkey, cv. Red Aleppo from Syria, and cultivars from the United States; (2) the Cultivar_II group, which includes 23 accessions mainly from Syria, Turkey, Greece, and Italy; and (3) the Cultivar_III group, which includes 107 accessions primarily from Iran, Turkey, and the United States (Figure 4A). The two major varieties Kerman and Peters that are widely cultivated in the United States are in the Cultivar_III and Cultivar_I groups, respectively. Population structure analysis revealed both population substructure and genetic heterogeneity in the Cultivar_I group (Figure 4C), indicating that there are many intra- and interspecific hybrids in Cultivar_I that have genomic components potentially introgressed from Cultivar_II and Cultivar_III as well as congener species. This result suggests continuous improvement of pistachio via hybridization with congener species after its domestication.

The nucleotide diversity (π) of the cultivated *P. vera* subgroups Cultivar_I, II, and III was estimated to be 0.93 × 10^−3^, 0.94 × 10^−3^, and 1.02 × 10^−3^, respectively, lower than that of the wild *P. vera* population (1.16 × 10^−3^) (Supplemental Figure 12A and 12B). The average Tajima’s D value for cultivated *P. vera* was estimated to be 1.247, much higher than that of wild *P. vera* (0.176). However, there are no apparent differences in the patterns of nucleotide diversity distributed along each pistachio chromosome. The average genetic divergence (*Fst*) between wild *P. vera* and the Cultivar_I, Cultivar_II, and Cultivar_III groups is 0.16, 0.18, and 0.21, respectively. A high Tajima’s D value in pistachio cultivars (average of 1.247) implies the effects of a population bottleneck in *P. vera* cultivars during or after domestication.

After purging accessions with potential interspecific admixture (as shown in Figure 4C) from each subgroup, our analysis of linkage disequilibrium (LD) decay showed delayed patterns for the three cultivar subgroups compared with the wild group (Supplemental Figure 12C). LD decay in Cultivar_I (10 accessions), Cultivar_II (20 accessions), and Cultivar_III (91 accessions) reached half of the r^2^ value at 21.63, 21.21, and 129.16 kb, respectively, whereas that of the wild population reached half of the r^2^ value at 3.96 kb. This pattern indicates that the three cultivated groups underwent a severe bottleneck during domestication. Alternatively, the numbers of individuals used for this analysis differed among subgroups, which may have affected the results.

#### Demographic history analysis shows population bottlenecks in pistachio

**Figure 5:**
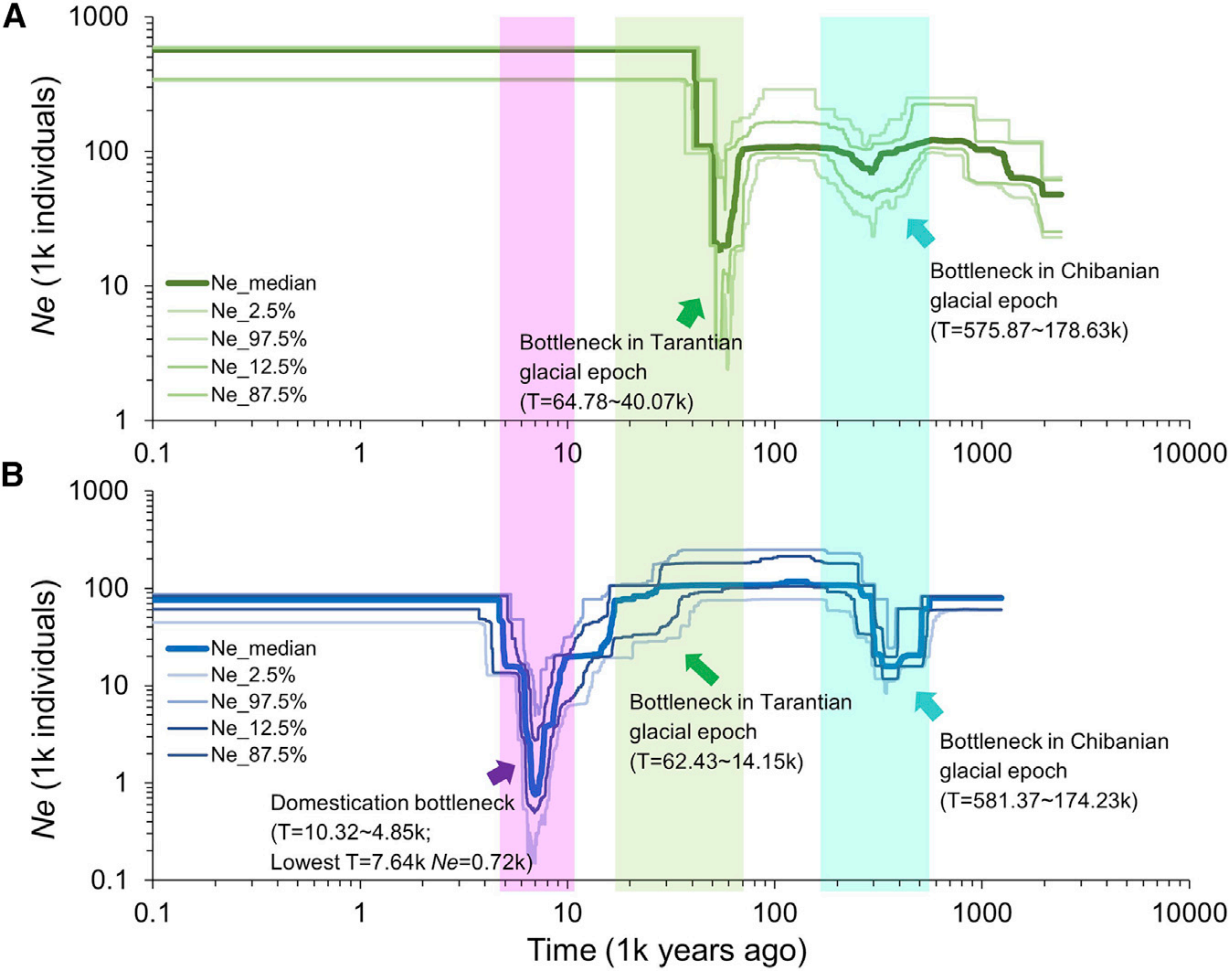
Demographic history analysis in pistachio Historical effective population size (*N*_*e*_) for wild **(A)** and domesticated (Cultivar_III subgroup) **(B)** pistachio populations. Stairway plot shows that the cultivated population has undergone one recent *N*_*e*_ decline caused by a domestication bottleneck at 10.32–4.85 (lowest at 7.64) Kya (purple). The wild population does not exhibit the domestication bottleneck but did experience two rounds of ancient geologic upheaval during the Chibanian at 575.87–178.63 Kya (green) and the Tarantian at 64.78–40.07 Kya (blue) in the last glacial epoch. The estimate is the median (thick line) of 200 bootstrap replicates with 2.5%, 12.5%, 87.5%, and 97.5% confidence intervals (four thin lines).

Demographic history analyses for the domesticated subgroup and wild population were performed using individuals without inter-group admixture for the Cultivar_I, Cultivar_II, and Cultivar_III subgroups and the wild population. Because the number of such individuals in Cultivar_I and Cultivar_II was insufficient for performing Stairway Plot analysis, the results show only the domesticated pistachio Cultivar_III subgroup and the wild population. Both populations had undergone bottlenecks during two periods: the first during the Chibanian at 575.87–178.63 Kya for the wild population and at 58.137–174.23 Kya for the domesticated population, and the second in the Tarantian after the last glacial epoch at 64.78–40.07 Kya for the wild population and at 62.43–14.15 Kya for the domesticated population (Figure 5A and 5B). The two ancient declines in *N*_*e*_ may have resulted from two rounds of temperature decreases during the last glacial cycle. Although the domesticated population exhibited these two ancient bottleneck events, a later drastic reduction (10.32–4.85 Kya) in *N*_*e*_ approximately 7640 years ago (Figure 5B), as described in other crop species such as maize and rice, was also detected in domesticated pistachio (Eyre-Walker et al., 1998; Meyer and Purugganan, 2013). Archaeological evidence indicates that pistachio nuts were being domesticated for food as early as 8770 years ago in Central Asia and the Middle East (Kashaninejad and Tabil, 2011). Our demographic analysis showed that the minimum *N*_*e*_ occurred close to this date, supporting the notion that cultivated pistachio underwent a severe domestication bottleneck approximately 7.64 Kya.

#### Genome-wide selective sweeps related to pistachio domestication

**Figure 6:**
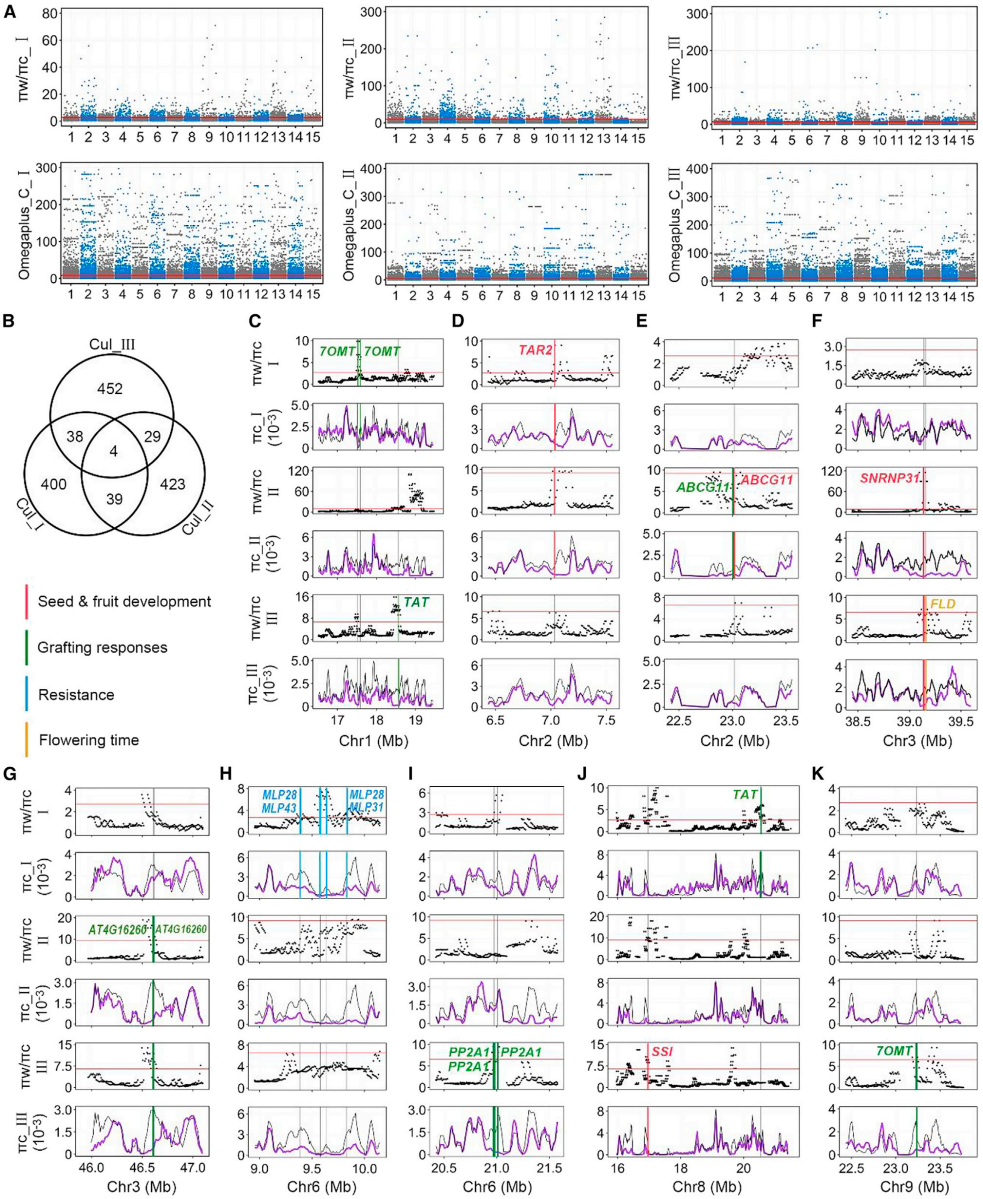
Domestication selective sweeps experienced by pistachio. **(A)** Manhattan plot shows the landscapes of selective sweeps in three subgroups, Cultivar_I, Cultivar_II, and Cultivar_III, detected using both π_Wild_/π_Cultivar_ ratio statistics and OmegaPlus scores across the female Siirt pistachio reference genome. The red solid line indicates the candidate regions identified above the 5% cutoff for each statistic. **(B)** Venn diagram of selective sweep genes detected by both statistics among the three subgroups. **(C–K)** Graphs showing genes that have undergone selective sweeps and are associated with agronomic traits (including seed and fruit development; grafting responses; resistance; and flowering time shift). The highlighted color signals show sweep genes identified in specific subgroups I, II, and III.

To identify potential selective sweeps, we scanned genomic regions for reduced nucleotide diversity in the cultivated groups (π_C_) relative to the wild groups (π_W_), measured as the π_W/π_C ratio in 50-kb sliding windows and 10-kb steps across the female genome for the three subgroups Cultivar_I, II, and III. The LD-based OmegaPlus (Alachiotis et al., 2012) scores were also used to detect selective sweeps using a grid size of 20 kb. We identified the intersecting regions of the top 5% of outlier regions for both statistics. The selective sweep regions were merged if outlier regions overlapped. We were able to detect 390, 211, and 290 potential selective sweeps in the Cultivar_I, II, and III subgroups, respectively. They averaged 31.29, 37.83, and 36.53 kb in length, occupied 1.99% (12.20 Mb), 1.30% (7.98 Mb), and 1.72% (10.59 Mb) of the assembled genome, and accounted for 1.62% (481 genes), 1.67% (495 genes), and 1.76% (523 genes) of the annotated genes (Figure 6A and 6B). There were 43 genes shared between Cultivar_I and II, 42 genes shared between Cultivar_I and III, and 33 genes shared between Cultivar_II and III, and four sweep genes were shared among the three subgroups (Figure 6B).

GO enrichment analysis showed that swept regions of Cultivar_I were enriched in the GOs reproductive process, seed development, hormone biosynthetic process, cell development, tissue development, and cell–cell junction, as well as fatty acid synthase (Supplemental Table 25A). Cultivar_II swept regions were enriched in the GOs organ morphogenesis, cell junction, immune system process, embryonic axis specification, seed coat development, and transferase activity. Cultivar_III swept regions were enriched in the GOs cellular response to external abiotic and wounding stimulus, gene silencing by RNA, terpene synthase, seed development, cellular response to water deprivation, and cell wall and extracellular region parts. The sweep genes shared between at least two subgroups were enriched in the GOs reproductive process, seed development, ethylene metabolic process, response to stimulus, and cell junction (Supplemental Table 25A). To further explore the genomic divergence between cultivars and wild populations, we identified genes in the sweep regions that potentially controlled agronomic traits (Figure 6C–6K).

#### Artificial selection improving pistachio zygote formation and seed development

Cultivated pistachio nuts are highly nutritious and rich in fatty acids, proteins, and starch stored in cotyledons and embryos. Several genes under artificial selection are related to zygote formation, embryogenesis, and seed development from fertilized zygote formation to the end of seed dormancy (Figure 6D–6F, 6J; Supplemental Table 25B). These include homologs of genes related to fertilization and zygote formation (*NFD4*, *ZAR1*); gynoecium, embryo, and endosperm development (*TAR2*, *STRAP*, *ABCG11*); and seed qualities related to fatty acid and starch synthesis (two *KCS* homologs; *SSI*), seed coat development (*ABCG11*, two *MYB5-like*), and seed maturation (*SNRNP31*).

Several genes that have undergone strong selective sweeps in pistachio have known functions in fertilization and zygote formation and ovule development in *Arabidopsis* (Figure 6D–6F, 6J; Supplemental Table 25B). *NFD4*, which encodes the *Arabidopsis* protein RPL21M, is required for fusions of nuclei that occur during development of the female gametophyte and double fertilization (Portereiko et al., 2006). *ZAR1* encodes a receptor protein kinase that acts during early zygote development to modulate asymmetric zygote division (Yu et al., 2016). *TAR2* (Figure 6D) is required for patterning of the gynoecium in *Arabidopsis* but is later expressed in the outer layers that will develop into the silique valves (Stepanova et al., 2008). *ABCG11* (Figure 6D) functions in lipid transport and is localized in a polar manner in the early epidermic cuticle between the embryo and endosperm or inner integumenta of the *Arabidopsis* seed coat (Panikashvili et al., 2010). *SNRNP31* (Figure 6F) causes embryo lethality when homozygous and defective seed maturation when heterozygous (Kim et al., 2010). In addition, several genes are related to seed oil biogenesis and accumulation (Supplemental Table 25B). There are two *KCS* genes, also known as *FAE*, which encode β-ketoacyl-CoA synthases that control the elongation of medium-chain (e.g., C16) fatty acids to long-chain (e.g., C26) fatty acids in *Arabidopsis* seeds (Lemieux et al., 1990; Jasinski et al., 2012). *SSI* (Figure 6J; Supplemental Table 25B) encodes starch synthase 1 in rice, which synthesizes starch in rice endosperm amyloplasts (Fujita et al., 2011).

#### Selective sweep for adaptation to grafting during domestication

Grafting in plants is an old propagation practice that is still important for shortening the juvenility periods of modern woody perennial crops (Melnyk and Meyerowitz, 2015). The key process for grafting success is the development of a chimeric graft union, which involves many physiological and molecular responses such as wounding repair, establishment of tissue connections, and polar growth of scions and rootstocks (Goldschmidt, 2014; Melnyk and Meyerowitz, 2015; Warschefsky et al., 2016). All pistachio trees in the germplasm collections included in this study are grafted trees, whereas the wild relatives are natural seedlings. Several genomic regions that showed selective sweeps included genes related to wound response and repair, hormone synthesis, and response factors (*7OMT*, *TAT*, *ABCG11*, and *4CLL9*); junction-union formation (*AT4G16260* and *PP2A1*); and control of meristematic tissue growth in shoots, hypocotyls, stems, and roots (*ATJ15* and *HSP70-5*) (Figure 6C, 6E, 6G, 6I–6K; Supplemental Table 25B).

At the beginning of the grafting process, hormone synthesis cascade pathways and wound response factors are triggered to regulate the physiological activities induced by wounding (Stegemann and Bock, 2009; Goldschmidt, 2014; Warschefsky et al., 2016). The selected regions included three *7OMT* genes (two of which are tandem repeats) and two *TAT* genes (Figure 6C, 6J, and 6K; Supplemental Table 25B), which respectively encode (R,S)-reticuline 7-O-methyltransferase and tyrosine aminotransferase; the latter catalyzes transamination leading to plant radical scavenger formation, and both are specifically induced by mechanical wounding (Lopukhina et al., 2001; Sandorf and Holländer-Czytko, 2002; Mishra et al., 2011, 2013). The protein encoded by *ABCG11* (Figure 6E; Supplemental Table 25B) may control transport pathways of cutin, wax, and suberin in response to wounding (Geisler et al., 2005; Panikashvili et al., 2010; Verdaguer et al., 2016; Do et al., 2018).

After wounding induction, a chimeric graft junction union forms, and a series of actions follow, including polar cell growth and the activation of cell volume enlargement, proliferation, and expansion. Two tandemly repeated *AT4G16260* genes clearly detected in Cultivar_II and Cultivar_III (Figure 6G; Supplemental Table 25B) encoded glucan endo-1,3-beta-glucosidase, which, together with endo-1,4-beta-glucanases, is required for cell wall modification. These genes may be involved in the formation of chimeric graft unions, as glucan endo-1,3-beta-glucosidase facilitates the reconstruction of cell walls at *Nicotiana*/*Arabidopsis* interfamily graft junctions (Notaguchi et al., 2020). We also detected several *PP2A1* genes (Figure 6I; Supplemental Table 25B) that encode filament-forming structural phloem proteins in *Arabidopsis* and are specifically expressed in the phloem companion cell–sieve element complexes that cross the graft interface between scion and rootstock (Golecki et al., 1998; Dinant et al., 2003).

Finally, meristematic tissue growth in shoots, hypocotyls, stems, and roots is reconstructed after the formation of the graft junction. In *Arabidopsis*, *ATJ15* (Supplemental Table 25B) encodes a chaperone DnaJ homolog that is highly expressed in the root cap and root tip meristematic and elongation zones, as well as modulating lateral auxin transport (Boonsirichai et al., 2003). We also identified two *HSP70-5* homologs (Supplemental Table 25B). In grafted cucumber, abscisic acid (ABA) induces the expression of the *HSP70* gene under high temperatures (Li et al., 2014).

#### Sweep genes related to flowering time regulation and drought and stress resistance

The *Arabidopsis* ortholog of a gene we identified in pistachio, *FLD* (*FLOWERING LOCUS D*) (Figure 6F; Supplemental Table 25B), likely encodes a histone demethylase that can induce flowering and block the vegetative-to-reproductive transition (He et al., 2003). During pistachio domestication, this gene underwent selection, which may have shifted the flowering times of cultivars relative to those of their wild populations. Because pistachio is highly tolerant of drought (Esmaeilpour et al., 2015) and saline soils or water, it is often grown under such conditions (Sheibani, 1995). We found four MLP-like protein genes, two *MLP28s*, *MLP43*, *MLP31* (Figure 6H; Supplemental Table 25B), which may be responsible for pistachio drought resistance. In *Arabidopsis*, the MLP-like protein regulates drought-stress responses via ABA signaling by regulating water loss (Wang et al., 2016). Another gene, *LRK10L-2.4* (Supplemental Table 25B), encodes a protein similar to a receptor-like kinase that may also influence ABA and drought-stress responses (Lim et al., 2014). Several other genes may be involved in disease responses or resistance (Supplemental Table 25B). These include *AIG1*, *RGA3*, and *At4g27220* (Reuber and Ausubel, 1996; Van Der Vossen et al., 2003; Kohler et al., 2008).

## Discussion

To understand the landscape of pistachio ZW sex chromosome evolution and the genomic architecture of the SDR on the W-chromosome, we sequenced and assembled the female and male pistachio genomes using PacBio long reads with Hi-C technology. The pistachio sex chromosomes were assembled as Z and W homologous chromosomes. Our definitions of the putative SDR and W-specific region and identification of candidate sex determination W-specific genes were sufficiently supported by multiple lines of evidence.

The large (12.70-Mb) non-recombining SDR region currently defined may be larger than the actual SDR region. The currently defined SDR is three times larger than its Z counterpart, which may be an artifact. Based on the current evidence of collinearity between the SDR and the ZZ genome, four regions may potentially have been translocated from either pseudo-autosome or autosome regions. The W chromosome in pistachio originated from an autosome following the first inversion at approximately 8.18 Mya, comparable with the origin of the sex chromosomes in *Silene latifolia* about 10 Mya (Bergero et al., 2007) and earlier than those in papaya approximately 7 Mya (Wang et al., 2012a; 2012b) and in spinach about 1.98 Mya (Ma et al., 2022). Three inversions in the W chromosome, followed by four potential translocation events, formed a large non-recombining region of 12.70 Mb. This finding is consistent with the evolutionary strata theory of sex chromosome evolution via several ordinal inversion events, as observed in human (Lahn and Page, 1999), chicken (Handley et al., 2004), *S. latifolia* (Bergero et al., 2007), and papaya (Wang et al., 2012b). The W-specific sequences in this study contain several genes of interest, especially *DYT1*, six *VPS13As,* and two *PTEN1s,* as well as *defA* and *defA*-like genes, which are present in all resequenced female genomes and absent in all examined male genomes. These genes may have played a pivotal role in the sex determination and/or differentiation of dioecious pistachio and may have contributed to the development of its ZW sex chromosome system during evolution.

Currently, we cannot conclude that the Middle East, including the Sarakhs area of Iran, is the center of domestication of pistachio based upon the population genomics and geographic distribution of wild and cultivated *P. vera*, as we currently have wild accessions only from the Sarakhs region. A high Tajima’s D value in cultivated *P. vera* implied population bottlenecks in *P. vera* cultivars after domestication. Delayed patterns of LD decay in cultivated *P. vera* compared with the wild population further indicated that cultivated *P. vera* underwent a bottleneck during domestication. Demographic history analysis showed that the wild pistachio population underwent two bottlenecks and supported a date for pistachio domestication at approximately 7.64 Kya, close to the estimate (8.77 Kya) from the archeological record (Kashaninejad and Tabil, 2011) and to that of a population genomic analysis (8.00 Kya) (Zeng et al., 2019).

Selective sweeps during the domestication of pistachio have affected several agronomic traits, such as nut and fruit development, grafting success, resistance, and flowering time shift. Pistachio nuts acquired high nutritional content in the form of fatty acids, proteins, and starch during the cultivation of nut trees from the wild. We found selective sweep genes related to zygote formation (such as *ZAR1*), seed development (such as *TAR2* and *ABCG11*), and seed nutrition accumulation (*KCS* and *SSI*) that may have contributed to the development of highly nutritious pistachio nuts due to human domestication (Stepanova et al., 2008; Panikashvili et al., 2010; Fujita et al., 2011; Jasinski et al., 2012; Yu et al., 2016). The grafting of a scion onto a rootstock is a regular practice for clonal production of a cultivar in pistachio orchards. The success of grafting involves many physiological and molecular responses (Goldschmidt, 2014; Melnyk and Meyerowitz, 2015; Warschefsky et al., 2016). Selective sweeps included many genes related to wound responses and repair and junction-union formation related to grafting. These genes have undergone strong selective sweeps and may also have been fixed by artificial selection. Although pistachio adapts very well to drought conditions, drought affects vegetative and reproductive processes and causes a reduction in yield and nut quality characters. We found evidence for artificial selection on several genes encoding MLP-like proteins, which influence drought-stress responses through regulation of water loss (Wang et al., 2016).

## Methods

### Part 1. Genome sequencing, assembly, and annotation

#### Plant materials, library construction, and sequencing

We conducted whole-genome sequencing for *P. vera* cv. Siirt (female) and cv. Bagyolu (male). A total of 67 *P. vera* and 16 wild *Pistacia* accessions were resequenced at ∼10× coverage for population genomics analyses (Supplemental Table 26). In addition, 142 (93 domesticated and 14 wild *P. vera* together with 35 other wild *Pistacia* species) resequenced accessions (Zeng et al., 2019) were used for population genomics analyses. A total of 125, 123, and 190 progenies from three F1 segregating populations between *P. vera* cv. Siirt and *P. vera* cv. Bagyolu (POP1), between cv. Siirt and Pa-18 (*Pistacia atlantica* Desf.) (POP2), and between cv. Ohadi and Pa-18 (POP3) were used for linkage mapping. DNA was extracted from fresh leaves of pistachio accessions and progenies using the CTAB method (Doyle and Doyle, 1987). To analyze the transcriptome of pistachio, several major tissues such as buds, flowers, shoots, and leaves from Siirt (female) and Atli (male) cultivars and whole nuts, kernels, and pericarps from cv. Siirt were sampled at different time points (Karcι et al., 2020). We extracted total RNA from these tissues using a modified CTAB protocol (Moazzam Jazi et al., 2015).

Multiple paired-end Illumina, mate pair Illumina, SMRTbell DNA, Hi-C, and CHICAGO libraries were constructed for cvs. Siirt and Bagyolu for sequencing. Details of library construction and sequencing are described in Supplemental Note 1. For high-density linkage map construction, Diversity Arrays Technology (DArT PL) and proprietary genotyping-by-sequencing (DArTseq) reduced-representation libraries were prepared as described by Kilian et al. (2012) and Sansaloni et al. (2011).

#### Construction of high-density linkage maps

A linkage map was constructed using the OCD MAPPING program from DArT PL (Petroli et al., 2012), which implements a marker-ordering algorithm combined with a tunable double crossover masking algorithm. Markers were clustered into linkage groups according to the method described by Wu et al. (2008). Markers with identical genotypes were placed in redundant bins, and the resulting markers/bins within each linkage group were ordered using the traveling salesman path solver program Concorde (Applegate et al., 2006). Apparent double-crossovers were masked before reordering the linkage groups and calculating recombination fractions. The Kosambi function was used to estimate genetic distances. A consensus linkage map was produced for each parent by combining the relevant SNP markers, resulting in common markers on each linkage map. The common markers were used to join all linkage maps into a single population consensus map using a linkage map as a seed map.

#### Overview of pistachio genome assemblies and annotation

The genome of cv. Siirt (female) was assembled by incorporating multiple technologies, including Illumina paired-end and mate pair libraries, PacBio long-read sequencing, and Dovetail Hi-C libraries. The Illumina-based assembly was constructed using SOAPdenovo2 (Luo et al., 2012) and SSPACE (Boetzer et al., 2011). To generate a better genome representation of pistachio, we generated ∼7 million PacBio long reads from a total of 68 SMRT cells, yielding 60.1 Gb of sequence (an estimated 90× genome coverage) with a median read length of 7 kb. We used the Celera Assembler (Berlin et al., 2015) to correct and assemble the raw PacBio reads, and we filtered heterozygous sequences based on an algorithm that improves contiguity for heterozygous genomes by popping “bubbles” inside the assembly graph (Supplemental Note 2). The draft PacBio assembly was then polished using QUIVER with all raw reads and further improved using the GATK pipeline (McKenna et al., 2010). SSPACE (Boetzer et al., 2011) was used to further scaffold the PacBio contigs with the 2-, 5-, 9-, and 20-kb mate pair libraries, requiring at least five pairs to join adjacent scaffolds.

For cv. Bagyolu (male), we generated ∼5.6 million PacBio long reads from a total of 52 SMRT cells, yielding 50.4 Gb of sequence (an estimated 76× genome coverage) with a median read length of 6.1 kb. CANU v1.7 (Koren et al., 2017) was used to assemble the PacBio reads with the parameter corOutCoverage = 200 to correct the PacBio reads. The 40× short reads were used to correct the top 36× long reads, and assemblies were performed with default parameters using 25× corrected reads as input. As the genome is highly heterozygous, we also used Redundans (Pryszcz and Gabaldón, 2016) to reduce heterozygous sequences with 50% minimum identity and 90% minimum overlap.

Chromosome-level assemblies of the Siirt and Bagyolu genomes were first achieved using Dovetail Hi-C technology, which used HiRise (Putnam et al., 2016) to anchor the sequences into 15 chromosomes. To improve the chromosome-level assemblies, ALLMAPS (Tang et al., 2015) was used to integrate Hi-C scaffolding and genetic linkage maps from three F1 populations. In brief, Hi-C superscaffolds generated by Dovetail Genomics (Scotts Valley, CA) were split into contigs simply by removing gaps. The ordering and orientation of contigs were recorded in the Hi-C map, which was treated as the input CSV file for ALLMAPS. In addition, six genetic linkage maps were uniquely anchored onto the contigs. The six linkage maps and the Hi-C map were integrated using ALLMAPS with default parameters (Tang et al., 2015). We further improved the female chromosome-level assembly using a reference-guided scaffolding strategy. The female scaffolds were first mapped against 28 male superscaffolds using minimap2 (Li, 2018) and further ordered and oriented using the RaGOO program (Alonge et al., 2019) with default parameters.

We used several programs to annotate the genome assemblies; these are described in more detail in Supplemental Note 3. We used the MAKER2 pipeline (Cantarel et al., 2008) to annotate protein-coding genes by integrating the data for assembled RNA-seq transcripts, homology-based gene prediction, and *ab initio* gene prediction. The annotation was then assessed using BUSCO (Simão et al., 2015). Details regarding the prediction of repetitive elements, non-coding RNAs, and tRNAs, identification and classification of NBS-encoding genes, analysis of alternatively spliced (AS) genes, and prediction of protein subcellular locations are provided in Supplemental Note 3.

### Part 2. Sex chromosome analyses

#### Identification of the pistachio sex chromosome

The ZW sex chromosome was identified from evidence of recombination suppression along female chromosomes. The correspondences between the physical and genetic map positions for each of the six genetic maps were first reconstructed by blastN searches of the sequences of genetic maps in both the female Siirt genome and male Bagyolu genome assemblies. The correlation curves between physical (Mb) and genetic (cM) distances in both genomes for each of the six genetic maps were plotted. A chromosome that showed low correlation between genetic and physical distances indicative of recombination suppression was identified as a candidate Z/W chromosome.

#### Identification of the pistachio SDR

To further define the SDR in the ZW chromosome, we compared the read coverages between pooled resequenced genomes of females (nine individuals from F1 population) and males (nine individuals from F1 population), then calculated the ratios of read coverage between the two sexes (F/(F + M) and M/(F + M)). Regions with reduced or missing male reads (F/(F + M) > 0.5; M/(F + M) < 0.5) compared with the genome-wide read coverage ratio (cutoff threshold F/(F + M) = 0.5 and M/(F + M) = 0.5) represented W-specific regions. Regions with higher read coverage (F/(F + M) < 0.5; M/(F + M) > 0.5) or equal read coverage (F/(F + M) ≈ 0.5; M/(F + M) ≈ 0.5) in males compared with females represented the W counterpart on the Z chromosome. The boundaries of the SDR in pistachio were defined by the region that showed continuous divergent read coverage between the two sexes.

In addition, the following evidence was also used for defining and verifying the SDR. GWAS of the two sexual phenotypes (22 resequenced females and 22 resequenced males) was performed using the EMMAX method (Zhou and Stephens, 2012). EMMAX was performed with parameters d = 10, v = verbose mode to generate a kinship matrix, and association analysis was implemented with population structure as the covariate. The genetic differentiation (*Fst*) between the sexes, as well as female-specific SNPs based on the data, were calculated using VCFtools (Danecek et al., 2011). We screened the region associated with the top GWAS score of −log10(*P*) ≥ 10, the top 1% value of *Fst* in each 20-kb window, and the continuous blocks of female-specific SNPs. The detailed boundaries of the SDR were then confirmed jointly using the above evidence and further defined using the collinear relationships between W and Z gene pairs.

#### Collinear analysis of the SDR and its Z counterpart

MCScanX (Wang et al., 2012a) was used with default parameters to detect collinear blocks between gene models in the previously identified putative SDR and its Z counterpart. The male gene models were used as the database and the female gene models as the query for MCScan searches, with an e value ≤ 1e−10. The microsynteny implemented in MCScanX was used to detect rearrangement events between the SDR and its Z counterpart by analysis of Z and W gene pairs.

#### Estimation of the divergence of gene pairs between SDR and its Z counterpart

The gene pairs in Z and W chromosomal regions were sequentially aligned using ClustalW2 (McWilliam et al., 2013). We estimated substitution rates at synonymous (*Ks*), non-synonymous (*Ka*), and silent (*Ksil*) sites following the Nei and Gojobori method in DnaSP v5 (Rozas et al., 2003). We determined divergence times for gene pairs using a molecular clock rate for the *Ksil* estimates of mu = 7.5 × 10^−10^, as previously estimated for the genus *Pistacia* (Parfitt and Badenes 1997).

#### Estimation of repetitive elements in SDR and its Z counterpart

Repeat sequences were predicted using custom *de novo*-assembled repeat libraries of the female and male genomes using RepeatModeler (http://www.repeatmasker.org/RepeatModeler/). We identified and clustered repetitive elements using consensus TE sequences imported into RepeatMasker v4.05 (Smit et al., 2013) and further classified unknown TEs by analyzing them in TEclass v2.1.3 (Abrusán et al., 2009). We identified tandem repeats using the Tandem Repeat Finder (TRF) package v4.07 (Benson, 1999) with the modified parameters “1 1 2 80 5200 2000 –d -h”.

#### Identification of SDR-specific blocks and genes

The SDR-specific blocks/genes were retrieved from the MCScanX results and defined as W genes with no homologs in the Z counterpart, whereas the Z counterpart-specific blocks/genes were identified as Z genes with no homologs in the W SDR. To narrow down the identified SDR-specific blocks/genes and Z counterpart-specific genes, we used reciprocal Blast searches between Z/W and Z chromosomes to identify W-specific and Z-specific genes. We identified the sequences that aligned between the two if they met the criteria of 99% identity and 1000 matching base pairs; we then retained the remainder as W-specific sequences if they did not have a hit in the Z chromosomes and as Z-specific genes if Z genes did not have a hit in the Z/W chromosomes. Finally, the intersecting results identified by MCScan and reciprocal Blast searches were treated as the final SDR-specific genes and Z counterpart-specific genes. Regions with reduced/missing male reads (F/(F + M) > 0.5; M/(F + M) < 0.5) between the two sexes that also overlapped with SDR-specific genes were regarded as the final W-specific blocks/sequences.

#### GO enrichment of W-specific genes reveals candidate sex determinant genes

GO functional annotation was performed in eggNOG-mapper v4.1 (Huerta-Cepas et al., 2017) through orthology assignment. GO term enrichment analysis was performed for W-specific genes with female gene models as references. We used Fisher’s exact test to determine the significance of GO-term enrichment. W-specific genes whose homologous genes function in floral organ development and regulation were considered to be potential candidates for W-encoded sex determinants.

### Part 3. Resequencing and population genomics analysis

#### Sample collection, sequencing, and variant calling

Genomic DNA was extracted from leaf tissues of 83 *Pistacia* accessions (Supplemental Table 26) using the Qiagen DNeasy Plant Mini Kit. Genomic libraries were constructed for 150-bp paired-end sequencing using the NEBNext Ultra DNA Library Prep Kit and sequenced using the Illumina HiSeq 2500 platform to generate raw paired-end Illumina reads. Also, 142 resequenced *Pistacia* genomes from a published paper were included for downstream analysis (Zeng et al., 2019).

The raw paired-end reads from 225 resequenced *Pistacia* genomes were trimmed using Trimmomatic (Bolger et al., 2014) after quality control with FastQC (Andrews, 2010). We used Bowtie 2 (Langmead and Salzberg, 2012) with default parameters to map trimmed reads to the female cv. Siirt genome. We then used the Genome Analysis Toolkit (GATK) (McKenna et al., 2010) to call variants with HaplotypeCaller using the default parameters. A total of 37 183 124 unfiltered variants (SNPs and InDels) were obtained. Variants with DP < 2 or DP > 40, minQ <20, >20% maximum-missing rate, or minor allele frequency (MAF) < 5% were removed from the raw VCF data. By filtering the data in these ways, we were able to reduce the number of variants for subsequent analyses to 5 368 583 SNPs and Indels. We used SnpEff v3.6c (Cingolani et al., 2012) to annotate the effects of variants including SNPs, Indels, and other synonymous or nonsynonymous variants, intronic variants, and variants located in the upstream or downstream regions of genes or in intergenic regions.

#### Analyses of genomic diversity, PCA, phylogeny, and population structure

We used the filtered set of 5 368 583 variants to calculate genomics statistics for the populations. We calculated SNP densities, π, Tajima’s D, and *F* statistics (Weir and Cockerham *Fst*) from the filtered data in the VCF file in a 50-kb sliding window with 10-kb steps in VCFtools (Danecek et al., 2011). After purging accessions with potential interspecific admixture (shown in Figure 4D) from the Cultivar_I (10 accessions), II (20 accessions), and III (91 accessions) subgroups, LD was calculated and its decay curve fitted for each subgroup in PopLDdecay (https://github.com/BGI-shenzhen/PopLDdecay).

Because the pistachio genome contains an SDR, we excluded the sex chromosome from downstream analyses. We used GCTA (Yang et al., 2011) to perform a PCA. We used VCFtools (Danecek et al., 2011) and PLINK (Purcell et al., 2007) to convert the VCF file into Plink binary files. We then used the top 2 principal components to assign the 225 pistachio accessions. We used 4 976 299 SNPs that were either bi-allelic or polymorphic to reconstruct a phylogeny of the pistachio accessions using SNPhylo (Lee et al., 2014). We used ADMIXTURE (Alexander et al., 2009) to infer ancestral population stratification for 174 pistachio accessions with ancestral population sizes K = 1–30 and chose the optimal population size as that with the least error after cross-validation.

#### Estimation of demographic history

The site frequency spectra (SFS) of cultivated and wild *P. vera* accessions were estimated using ANGSD (Korneliussen et al., 2014). We used the filtered BAM files generated from mapping reads from the pistachio accessions to calculate site allele frequencies at all sites using the genotype likelihood model in SAMtools. We then used the Expectation Maximization algorithm to compute a maximum likelihood estimate of the folded SFS. The SFS was then used to estimate population demographic history using Stairway plots (Liu and Fu, 2015) with 200 bootstrap iterations. Because of variation in the molecular substitution rate and generation time among the Anacardiaceae (Parfitt and Badenes, 1997), we used a range of molecular clocks (6e^−9^, 8e^−9^, and 10e^−9^ per site per generation) as mutation rate parameters and generation times of 6, 8, and 10 years to estimate the demographic history of pistachio.

#### Detection of domestication selection

In terms of genome-wide selection, we detected artificial selective sweeps by comparing nucleotide diversity between cultivated and wild populations, excluding highly admixed accessions from our analyses. We would expect genomic regions and genes that have undergone domestication sweeps in cultivated crops to exhibit significantly lower nucleotide diversities than corresponding regions in their wild pistachio relatives. We determined the ratio of genetic diversity (π_W/π_C) between the wild population and each of three cultivated pistachio groups (Cultivar_I, Cultivar_II, Cultivar_III) by comparing their nucleotide diversities in 50-kb sliding windows with 10-kb steps. The candidate regions were defined as the top 5% of π_W_/π_C_ statistics, including 4-kb flanking regions on both sides.

To detect the selective sweeps with greater confidence, we also used LD-based OmegaPlus software (Alachiotis et al., 2012) to narrow the selective sweep regions in the cultivated groups using a 20-kb grid. The top 5% OmegaPlus score outlier regions and 4-kb flanking regions on both sides were also regarded as part of the candidate sweep regions. The intersecting regions detected by both statistics were selected and merged if the outlier regions overlapped. Finally, each set of overlapping windows was merged into a single selected region. Genes that overlapped with swept regions were treated as putatively under selection. The functions of selective sweep genes were then annotated by blasting against the NCBI NR database (ftp://ftp.ncbi.nih.gov/blast/db).

## Funding

The authors would like to thank the 10.13039/501100004410Scientific and Technological Research Council of Turkey (project nos. TUBITAK-TOVAG 100 O 113 and TUBITAK-TOVAG 113 O 962), the University of Çukurova Scientific Research Projects Unit (project nos. FDK-2015-3641, FDK-2015-3642, FBA-2015-4521, FBA-2015-4538, FBA-2016-5406, FBA-2016-5442, FBA-2016-5407, FDK-2017-9232, FBA-2017-8250, and FBA-2020-11957), the 10.13039/501100004837Ministerio de Ciencia e Innovación of Spain (project nos. AGL2009-09094 and RYC-2011-08653), the 10.13039/501100006393University of Granada (project no. PP2016-PIP13), and the 10.13039/501100003392Natural Science Foundation of Fujian Province, China (project nos. 2021J01142 and 2018J01606) for providing financial support for this research. The authors wish to thank the Gaziantep Pistachio Research Institute and University of Çukurova in Turkey for providing plant materials.

## Author contributions

S.K. conceived the pistachio genome project. S.K. and Ray Ming coordinated the research activities and designed the experiments. S.K., Ray Ming, Xingtan Zhang, H.T., Richard Michelmore, J.L., C.M.W., H.Ö., M.K., M.G., HtT, A.P., H.K., E.J., and W.P. contributed to the whole-genome sequencing and assembly. Xingtan Zhang, X.X., HtT, Xiaodan Zhang, R.N.-P., R.d.l.H., J.A.G.-Z., F.R., C.R.R., C.d.V.M., A.H.-W., X.J.M., S.K., and N.Ç. performed the annotations. S.K., H.G., M.K., E.Z.M., N.Ç., N.E.K., and A.K. processed the F1 progenies and constructed genetic linkage maps. X.M., Ray Ming, S.K., H.H., X.L., K.L., Q.H., and H.L. performed analyses of the sex determination region, sex chromosome evolution, and identification of sex determination genes. X.M., Ray Ming, S.K., R.N.-P., R.d.l.H., C.R.R., N.Ç., and H.K. contributed to generating whole-genome resequencing data and to the population genomic and domestication analysis. S.K., Ray Ming, X.M., Xingtan Zhang, HtT, R.N.-P., H.T., C.d.V.M., X.J.M., and Xiaodan Zhang wrote the manuscript. Ray Ming, S.K., Richard Michelmore, and X.M. edited the manuscript. All authors have read and approved the final manuscript.

## Accession numbers

Data supporting the findings of this work are available within the paper and its supplemental information files. The datasets generated and analyzed in this study are available from the corresponding authors. All sequences used in this study have been submitted to the NCBI BioProject and Sequence Read Archive database under accession ID PRJNA680201. The whole-genome sequences of Siirt and Bagyolu have been deposited at NCBI under accession ID PRJNA625479.

## Supplemental information

Document S1. Supplemental Tables 1–26

Document S2. Supplemental Notes 1–3 and Supplemental Figures 1–12
