# Supplementary material for: Pistachio genomes provide insights into nut tree domestication and ZW sex chromosome evolution
Source: Plant Commun. 2022 Nov 26;4(3):100497. doi: 10.1016/j.xplc.2022.100497 (PMC10203267; doi:10.1016/j.xplc.2022.100497)
Supplement: Document S2. Supplemental Notes 1–3 and Supplemental Figures 1–12 [file mmc2.docx]

**SUPPLEMENTARY INFORMATION**

**The Pistachio Genomes Provide Insights into Nut Tree Domestication and ZW Sex Chromosome Evolution**

Salih Kafkas^1,20*^, Xiaokai Ma^2,3,20^, Xingtan Zhang^2^, Hayat Topçu^1^, Rafael Navajas-Pérez^4^, Ching Man Wai^5^, Haibao Tang^2^, Xuming Xu^2,6^, Mortaza Khodaeiaminjan^1^, Murat Güney^1^, Aibibula Paizila^1^, Harun Karcı^1^, Xiaodan Zhang^5^, Jing Lin^2^, Han Lin^2^, Roberto de la Herrán^4^, Carmelo Ruiz Rejón^4^, Jerson Alexander García-Zea^4^, Francisca Robles^4^, Coral del Val Muñoz^7,8^, Agnes Hotz-Wagenblatt^9^, Xiangjia Jack Min^10^, Hakan Özkan^11^, Elmira Ziya Motalebipour^1^, Hatice Gozel^12^, Nergiz Çoban^12^, Nesibe Ebru Kafkas^1^, Andrej Kilian^13^, HuaXing Huang^2^, Xuanrui Lv^2^, Kunpeng Liu^2^, Qilin Hu^2^, Ewelina Jacygrad^14^, William Palmer^14^, Richard Michelmore^14^, Ray Ming^5*^

**ADDITIONAL FILE 2**

**The Pistachio Genomes Provide Insights into Nut Tree Domestication and ZW Sex Chromosome Evolution**

Salih Kafkas^1,20*^, Xiaokai Ma^2,3,20^, Xingtan Zhang^2^, Hayat Topçu^1^, Rafael Navajas-Pérez^4^, Ching Man Wai^5^, Haibao Tang^2^, Xuming Xu^2,6^, Mortaza Khodaeiaminjan^1^, Murat Güney^1^, Aibibula Paizila^1^, Harun Karcı^1^, Xiaodan Zhang^5^, Jing Lin^2^, Han Lin^2^, Roberto de la Herrán^4^, Carmelo Ruiz Rejón^4^, Jerson Alexander García-Zea^4^, Francisca Robles^4^, Coral del Val Muñoz^7,8^, Agnes Hotz-Wagenblatt^9^, Xiangjia Jack Min^10^, Hakan Özkan^11^, Elmira Ziya Motalebipour^1^, Hatice Gozel^12^, Nergiz Çoban^12^, Nesibe Ebru Kafkas^1^, Andrej Kilian^13^, HuaXing Huang^2^, Xuanrui Lv^2^, Kunpeng Liu^2^, Qilin Hu^2^, Ewelina Jacygrad^14^, William Palmer^14^, Richard Michelmore^14^, Ray Ming^5*^

^1^Department of Horticulture, Faculty of Agriculture, University of Çukurova, 01330, Adana, Turkey,^2^Center for Genomics and Biotechnology, Haixia Institute of Science and Technology, School of Future Technology, Fujian Agriculture and Forestry University, Fuzhou, China, ^3^Key Laboratory of Orchid Conservation and Utilization of National Forestry and Grassland Administration, Fujian Agriculture and Forestry University, Fuzhou, China, ^4^Departamento de Genética, Facultad de Ciencias, Campus de Fuentenueva s/n, 18071, Granada, Spain, ^5^Department of Plant Biology, University of Illinois at Urbana-Champaign, Urbana, IL 61801, USA, ^6^Key Laboratory of the Ministry of Education for Coastal and Wetland Ecosystems, College of the Environment and Ecology, Xiamen University, Xiamen 361102, China, ^7^Department of Computer Science, University of Granada, Granada, Spain, ^8^Andalusian Research Institute in Data Science and Computational Intelligence (DaSCI Institute), 18014, Granada, Spain, ^9^German Cancer Research Center, Omics IT and Data Management Core Facility, Heidelberg, Germany, ^10^Department of Biological Sciences, Youngstown State University, Youngstown, OH 44555, USA, ^11^Department of Field Crops, Faculty of Agriculture, University of Çukurova, 01330, Adana, Turkey, ^12^Pistachio Research Institute, 27060, Şahinbey, Gaziantep, Turkey, ^13^Diversity Arrays Technology, University of Canberra, Canberra, Australia, ^14^Genome Center, University of California Davis, 451 Health Sciences Drive, Davis, CA 95616, USA

^20^These authors contributed equally.

*Corresponding authors

**SUPPLEMENTARY CONTENTS**

SUPPLEMENTARY NOTE 1. Library Construction and Sequencing

SUPPLEMENTARY NOTE 2. Genome Assemblies of Two Pistachio Cultivars

SUPPLEMENTARY NOTE 3. Annotations

SUPPLEMENTARY FIGURES (from Figure S1 to Figure S12)

SUPPLEMENTARY REFERENCES

**SUPPLEMENTARY NOTE 1**

**Library Construction and Sequencing**

Multiple paired-end Illumina libraries at insert sizes of 250 bp, 500 bp, and 800 bp and mate-pair libraries at insert sizes of 2 kb, 5 kb, 10 kb, 20 kb, and 40 kb were constructed. Sequencing of Illumina libraries was performed on a HiSeq 2500 platform to generate 303.3 Gb of sequence. To obtain clean reads, reads were filtered to remove low quality reads, adapter contamination, and reads with ambiguous bases (>10% Ns). A total of 187.6 Gb of clean data was retained for assembly.

SMRTbell DNA library preparation and sequencing were conducted in accordance with the manufacturer's protocols (Pacific Biosciences) using P6-C4 chemistry. Approximately 15 µg of high-quality genomic DNA was applied for size selection on the BluePippin (Sage Science) and SMRTbell libraries with 20-kb inserts were prepared. The genomes of the pistachio cvs. Siirt and Bagyolu were sequenced employing 68 and 52 Single-Molecule Real-Time (SMRT) cells on the PacBio RSII platform (Pacific Biosciences), respectively.

Two Hi-C libraries for cv. Siirt and one CHICAGO library and one Hi-C library for cv. Bagyolu were prepared by Dovetail Genomics. In cv. Bagyolu, a CHICAGO library was prepared as described previously (Putnam et al., 2016). Briefly, ~500 ng of HMW gDNA (~100 kb mean fragment size) was reconstituted into chromatin *in vitro* and fixed with formaldehyde for construction of one CHICAGO library. Fixed chromatin was digested with *Dpn*II, the 5’ overhangs filled in with biotinylated nucleotides, and the free blunt ends were then ligated. After ligation, crosslinks were reversed and the DNA was purified from proteins. Purified DNA was treated to remove biotin that was not internal to ligated fragments. The DNA was then sheared to a mean fragment size ~350 bp and sequencing libraries were generated using NEBNext^®^ Ultra enzymes and Illumina-compatible adapters. Biotin-containing fragments were isolated using streptavidin beads before PCR enrichment of each library. Dovetail Hi-C libraries were prepared in a similar manner in both cultivars as described previously (Lieberman-Aiden et al., 2009). The libraries were sequenced to generate 105 M (CHICAGO) and 78 M (Hi-C) 150 bp read pairs for cv. Bagyolu and 138 M and 74 M read pairs for cv. Siirt on the Illumina HiSeq platform. Sequencing of CHICAGO and Hi-C libraries in cv. Bagyolu provided 116.4X and 267.7X physical coverage of the genome, respectively, while two Hi-C library reads provided 2,044.5X physical coverage of the cv. Siirt genome (1–50 kb pairs).

To analyze the transcriptome of pistachio, the purity and quantity of each RNA sample were determined using a Qubit fluorometer, gel electrophoresis, and an Agilent Bioanalyzer 2100 prior to deep sequencing. Extracted RNA samples were treated with RNase-free DNase I to remove DNA contamination. Prior to cDNA library construction, mRNAs were isolated from total RNAs using the oligo (dT) method. Then the mRNAs were broken into short fragments of approximately 160 bp, and first-strand cDNA and second-strand cDNA were synthesized. cDNA fragments were purified and resolved with EB buffer for end repair and single nucleotide A (adenine) addition. The cDNA fragments were linked with adapters. The cDNA fragments of suitable size were selected for PCR amplification. An Agilent 2100 Bioanalyzer was used to quantify and assess the quality of those libraries. The cDNA libraries were sequenced on the Illumina HiSeq 2500 platform as 2 × 100 run. The low-quality sequence reads (more than 20% of the base qualities were below 10), adaptor-contaminated reads, and reads with a high content of unknown bases (N bases more than 5%) were removed before downstream analyses. Approximately 37.5 Gb of clean data were obtained from sequencing samples derived from different organs and tissues of female Siirt and male Atli pistachio cultivars.

**SUPPLEMENTARY NOTE 2**

**Genome Assemblies of Pistachio Cultivars Siirt and Bagyolu**

***Genome Assembly of cv. Siirt***

For cv. Siirt (female), we sequenced multiple Illumina libraries, including paired-end and mate pair libraries. As a genome survey, we first counted the number of *K*-mers (*K* = 25) in the Illumina paired-end data to reveal the level of heterozygosity (Gnerre et al., 2011). An Illumina-based assembly was constructed using SOAPdenovo2 (Luo et al., 2012) and SSPACE (Boetzer et al., 2011). To generate a better genome representation of pistachio, we generated ~7 million long PacBio reads using 68 SMRT cells yielding a total of 60.1 Gb (an estimated 90X genome coverage) with a median read length of 7 kb. We corrected the raw PacBio reads to ~28.5X genome coverage using the PBcR pipeline (Berlin et al., 2015). The corrected PacBio reads were then assembled using Celera Assembler ver 8.3rc2 (Berlin et al., 2015) with the following options: *unitigger=bogart, consensus=pbutgcns, batOptions=-RS –CS, ovlErrorRate=0.10, cgwErrorRate=0.10, cnsErrorRate=0.10, obtErrorRate=0.08, utgGraphErrorRate=0.05,* and *utgMergeErrorRate=0.06*. During the assembly, we ran an algorithm that improves the contiguity of heterozygous genomes by popping the "bubbles” inside the assembly graph (https://github.com/tanghaibao/jcvi/blob/main/jcvi/assembly/ca.py). The draft PacBio assembly was then polished using QUIVER (https://github.com/PacificBiosciences/GenomicConsensus) with all raw reads. Polishing with QUIVER ensures that all bases are at least QV50 quality (base error rate < 0.001%) and each base is covered by at least five supporting reads.

After running the bubble popping algorithm, we classified the contigs in the final assembly as primary or associative contigs, for which the assembly graph switches from homozygous (linear portions of the graph) to heterozygous regions (indicated as bubbles in the graph). The primary and associative contigs from the two sides of the bubbles in the assembly graph were given in Supplementary Note 2 Figure 1a,b. After evaluated by mapping of Illumina paired-end data, the primary and associative contigs were confirmed to have different read depth distribution (Supplementary Note 2 Figure 2a,b). The associative contigs show a single peak at lower read depth compared to the primary contigs, which contain a mixture of homozygous (two-alleles) and heterozygous (single-allele) regions, at high and low read depth, respectively.

We used SSPACE (Boetzer et al., 2011) to provide further scaffolding of the PacBio contigs with 2 kb, 5 kb, 9 kb, and 20 kb mate pair libraries, requiring at least five pairs to join adjacent scaffolds. Because PacBio contigs can contain a high number of InDel errors (Sakai et al., 2015) we further corrected the contig sequences using Illumina paired-end reads. To identify the InDel errors, we mapped the Illumina reads to the PacBio contigs using BWA-MEM (Li and Durbin, 2010). After local realignment and discarding PCR duplicates, we identified InDels using the HaplotypeCaller in the GATK package (Depristo et al., 2011) and selected only high-quality InDels using VariantFiltration with the following expression: *--filterExpression* ”*DP < 10 || DP > 300 || QD < 2.0 || FS > 60.0 || MQ < 40.0*”. In addition, we used *SelectVariants* to select only homozygous InDels: *-selectType INDEL --excludeFiltered -select* ”*vc.getGenotype("sm").isHomVar()*”*.* We corrected a total of 36,071 indels in the PacBio sequences with Illumina reads using *FastaAlternateReferenceMaker* in GATK. After polishing with Illumina reads, redundant contigs (98% coverage at 98% identity) and low complexity contigs (98% masked by DUSTMASKER) were removed from the assembly.

The input *de novo* assembly, shotgun reads, and Dovetail Hi-C library reads were used as input data for HiRise, a software pipeline designed specifically for using proximity ligation data to scaffold genome assemblies (Putnam et al., 2016) Shotgun and Dovetail Hi-C library sequences were aligned to the draft input assembly using a modified SNAP read mapper (<http://snap.cs.berkeley.edu>). The separations of Dovetail Hi-C read pairs mapped within draft scaffolds were analyzed by HiRise to produce a likelihood model for genomic distance between read pairs, and the model was used to identify and break putative misjoins, to score prospective joins, and make joins above a threshold. After scaffolding, shotgun sequences were used to close gaps between contigs. Scaffolding with Hi-C libraries made 167 breaks and 2,359 joins to the contig assembly. Additionally, we used the Hi-C libraries to detect 61 breaks and 660 joins. To improve the chromosome-level assemblies of cv. Siirt, ALLMAPS (Tang et al., 2015) was used to integrate Hi-C scaffolding and genetic linkage maps from three F1 populations. Briefly, Hi-C super-scaffolds generated from Dovetail Genomics (California, USA) were split into contigs simply by removing gaps. The ordering and orientation of contigs were recorded in the Hi-C map, which was treated as the input CSV file for ALLMAPS. In addition, six genetic linkage maps were uniquely anchored onto the contigs. The six genetic maps as well as the Hi-C map were integrated using ALLMAPS with default parameters.

***Genome Assembly of cv. Bagyolu***

For cv. Bagyolu (male), we generated ~5.6 million PacBio long reads from a total of 52 SMRT cells yielding a total of 50.4 Gb (an estimated 76X genome coverage) with a median read length of 6.1 kb. CANU v1.7 (Koren et al., 2017) was used to assemble the PacBio reads with parameter corOutCoverage=200, which means that all of the input PacBio reads will be corrected. The 40X short reads were used to correct the top 36X long reads, and assemblies were performed with default parameters with 25X corrected reads as input. As the genome is highly heterozygous, we further used Redundans (Pryszcz and Gabaldón, 2016) to reduce heterozygous sequences with 50% minimum identity and 90% minimum overlap, resulting in an assembly size of 622 Mb and contig N50 of 92 kb. Subsequently, the draft assembly was polished using the Pilon program (Walker et al., 2014) Two iterations of HiRise (Putnam et al., 2016) scaffolding were performed. First, shotgun and CHICAGO library sequences were aligned to the draft input assembly using a modified SNAP read mapper (<http://snap.cs.berkeley.edu>). The separations of CHICAGO read pairs mapped within draft scaffolds were analyzed using HiRise (Putnam et al., 2016) to produce a likelihood model for genomic distance between read pairs, and the model was used to identify and break putative misjoins, to score prospective joins, and make joins above a threshold. After aligning and scaffolding CHICAGO data, Dovetail Hi-C library sequences were aligned and scaffolded following the same method. After scaffolding, shotgun sequences were used to close gaps between contigs. Scaffolding with the CHICAGO library made 427 breaks and 964 joins to the contig assembly. We additionally used Hi-C libraries to detect four breaks and 404 joins. As an evaluation of assembly completeness for both genomes, we used a core set of conserved eukaryotic genes for CEGMA (Parra et al., 2007) and BUSCO (Simão et al., 2015) analyses.

**A.**

**
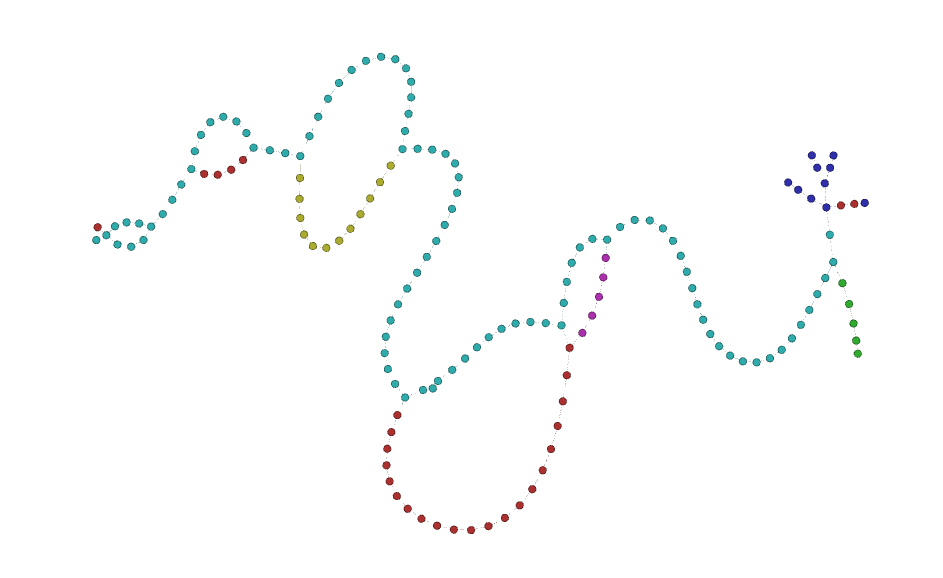
**

**B.**


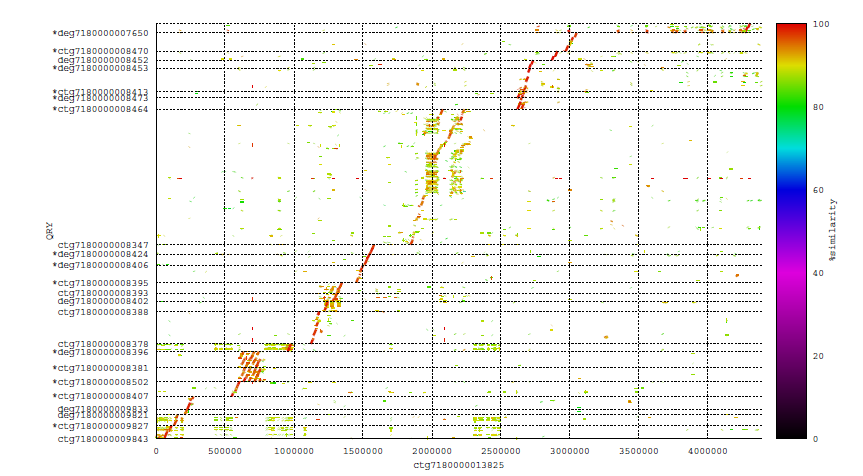


**Supplementary Note 2 Figure 1.** Bubbles in the pistachio assembly graph due to heterozygosity. (**A**) Assembly graph is represented by reads as nodes, read overlaps as edges. Contigs can be reconstructed by following paths within the assembly graph. Bubbles can be seen as paths that diverge and then reconverge. The two sides of a bubble represent paternal and maternal alleles. Color of the nodes represent distinct contigs reconstructed. The algorithm works by continuing an arbitrary path through several bubbles, with one primary contig and several associative contigs. (**B**) NUCMER alignments between the primary contig (*x*-axis) and several associative contigs (*y*-axis).

**A.**


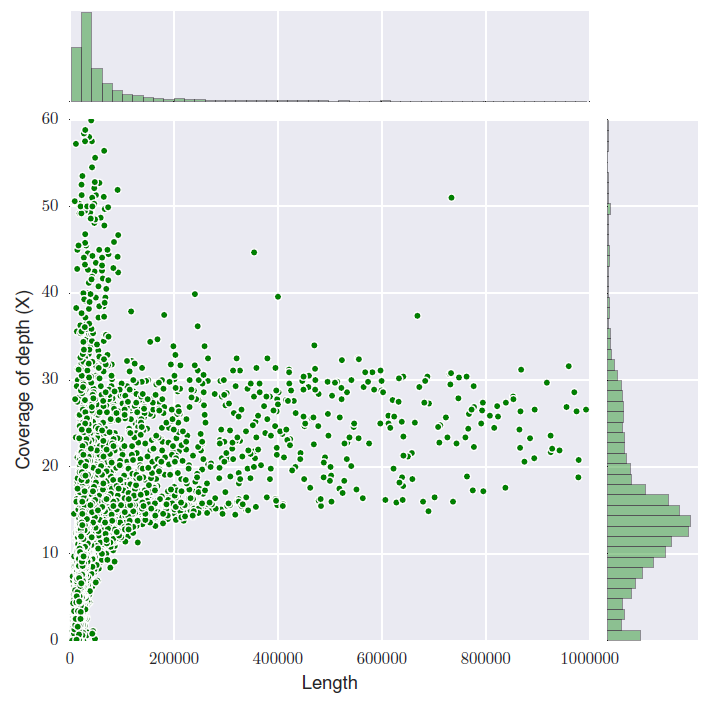


**B.**


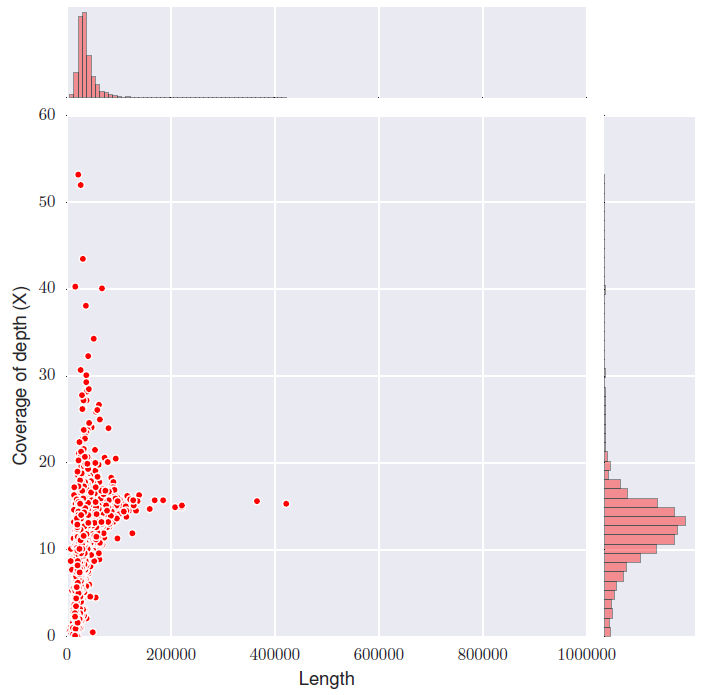


**Supplementary Note 2 Figure 2.** Depth of coverage for primary and associative contigs. (**A**) Primary contigs. (**B**) Associative contigs. The primary contigs contain a mixture of homozygous (two alleles) and heterozygous (single allele) regions.

**SUPPLEMENTARY NOTE 3**

**Annotations**

Comprehensive transcriptome assembly was carried out using both *de novo* TRINITY and reference-guided TRINITY (Grabherr et al., 2011), and the results were combined and used together as mRNA evidence for MAKER (Cantarel et al., 2008). MAKER was used for the gene annotation and each *ab initio* gene model was evaluated against matching transcript and protein evidence to select the model that was most consistent on the basis of the AED metric (Cantarel et al., 2008).

To generate high-quality annotation of protein-coding genes, we carried out two rounds of MAKER. Data input to MAKER was prepared as follows. First, *ab initio* gene predictors, including SNAP (Korf, 2004), GENEMARK (Lomsadze et al., 2005), and AUGUSTUS (Stanke et al., 2006) were each trained with ‘near full-length’ pistachio transcripts. The pistachio transcripts were constructed using PASA (Haas et al., 2008) and were evaluated against UNIPROT plant proteins to identify the set of near full-length candidates that cover at least 95% of any target protein. Plant proteins were downloaded from UNIPROT and used as protein evidence for MAKER. Putative proteins greater than 30 amino acids in length were retained. Intron locations were predicted based on alignments of RNA-Seq reads, and were used as hints to guide GENEMARK (Lomsadze et al., 2005) and AUGUSTUS (Stanke et al., 2006). MAKER was run on the pistachio assembly without repeat masking followed by extensive filtering of TE-related genes. In the second round of MAKER, the predicted gene models with an AED score less than or equal to 0.4 were extracted for re-training using AUGUSTUS. A total of 29,695 gene models were generated in the female pistachio genome and 29,996 genes were generated in the male pistachio genome.

BUSCO (Simão et al., 2015) version 3 was used to evaluate the completeness of annotations. In the female pistachio genome, 1,266 (87.9%) =of 1,440 conserved genes were re-annotated, and in the male pistachio genome, 1,004 (69.7%) of 1,440 conserved genes were re-annotated. For functional annotation, we used eggNOG 5.0 to interpret the potential functions of the protein-coding genes (Huerta-Cepas et al., 2017).

**Annotation of Non-Coding RNAs and tRNAs**

To annotate conserved miRNAs in pistachio, we used all mature miRNAs from mirBAse Release 22 (Kozomara and Griffiths-Jones, 2014) and all mature miRNAs from the plant microRNA Database (Zhang et al., 2009) as the query. These known miRNAs were mapped to the pistachio genome with Bowtie version 0.12.9 (Langmead et al., 2009) using the following parameters: -f -n 0 -l 14 -m 1 --best --strata -p 3 -S. Prior to mapping, the pistachio chromosomes were indexed for Bowtie with the default parameters. Mapping was performed with settings that did not allow for mismatches and only obtained unique hits. Those hits with that uniquely mapped to the genome were annotated as miRNAs in pistachio following the criteria described by Meyers et al. (Meyers et al., 2008). for miRNA annotation. If miRNAs could not be annotated because they mapped to multiple sites in the genome (Bowtie -f -n 0 -l 14 --all -p 3 -S for all hits), the multiple hits were further analyzed by looking for palindromes and RNA folding (EMBOSS:palindrome (palindrome -filter -minpallen=15 -maxpallen=50 -nummismatches=4 -gaplimit=150 -nooverlap) using Vienna Package 2.1.8:rnaLfold (RNALfold -z -L 150). Those miRNAs with clear evidence of conservation of both secondary stem loop structures and the mature miRNAs were recorded but not included in the final annotation. This analysis was carried out using our own custom scripts.

We predicted ncRNAs such as rRNAs, introns, ribozymes, or snoRNAs using Infernal and conducted inference of RNA secondary structure alignments with INFERNAL 1.1.2 (Nawrocki and Eddy, 2013) and the RFAM database version 14.0 (2,687 families) (Kalvari et al., 2018) over the sequences of the pistachio genome. The joint use of this search algorithm and the RFAM database has been shown to lead to a more complete annotation of RNAs in genomes than other methods (Nawrocki, 2014). We used the Infernal package to search with the RFAM “calibrated” covariance models (CM), which have a bit score threshold (also called gathering (GA) threshold) that allows for separation of the first clear false positive from trusted true homologs. All hits with bit scores above this threshold are considered to belong to a family. To reduce false positives to the minimum, the searches were performed using the --cut_ga option, which dictates that only hits exceeding the GA threshold will be reported. Results from RFAM were filtered to eliminate hits from different models that overlap by retaining those with the better E-value. If both hits have the same E-value, the one with the higher bit score was selected and included in the annotation.

Annotation of tRNAs was conducted using the tRNAscan-SE v2.0 program (Lowe and Eddy, 1997; Lowe and Chan, 2016) with the Eukarya covariance model and with default parameters over the pistachio male and female chromosomes and the chloroplast fasta files. tRNAscan-SE uses a powerful statistical model that accurately scores candidates based on both their sequence and predicted secondary structures. The generated results are comprised of tRNAs including those with introns and tRNA pseudogenes. Additionally, tRNAs were also predicted using Infernal version 1.1.1 (Nawrocki and Eddy, 2013) and the RFAM14.0 database over the sequences of the pistachio genome. Results from RFAM were filtered to eliminate hits from different models that overlap by keeping those with the better E-value, and if both hits have the same E-value, selecting the one with the higher bit score. Predictions from tRNAscan-SE and Infernal-RFAM were compared to remove redundant hits and annotate the remaining as pistachio tRNAs.

**Prediction and Analysis of Repetitive Elements**

The assembled chromosomes and scaffolds of female Siirt and male Bagyolu cultivars were separately analyzed with WindowMasker (Morgulis et al., 2006) to define low-complexity sequences and short repeats, and for the detection of interspersed repeats. The RepBase database was used (Bao et al., 2015) together with a custom library containing the repetitive elements from several plant genomes including rice, maize, *Arabidopsis*, *Phoenix*, *Ricinus, Vitis, Brassica*, and *Populus*. A two-step WU-BLAST experiment was then run. In the first run, all blastn hits against RepBase with an identity higher than 70% were masked. In the second run, ORFs were predicted using tblastn.

For more accurate detection of tandemly arrayed sequences, raw fastq Illumina paired-end sequences of cvs. Siirt and Bagyolu were filtered according to the following parameters: 100-bp length and quality Q>33. A total of 500K paired-end reads were randomly selected to run RepeatExplorer (Novák et al., 2010) with default options and a custom database of repeated sequences. Contigs were assembled using Cap3 (Huang and Madan, 1999) and consensus motifs were detected using Tandem Repeats Finder (Benson, 1999), dot-plot analysis by Geneious (https://www.geneious.com/features/sequence-alignment/), and by multiple alignment using Clustal X (Thompson et al., 1997). Consensus sequences were then blasted against the single chromosome sequences and mapped at high/medium sensitivity using Geneious. Clusters were considered tandemly arrayed when consensus sequences had at least five consecutive hits in the assembly.

**The Identification and Classification of NBS-Encoding Genes**

Using HMMER 3.1b2, protein sequences from the pistachio genome were scanned against the NBS domain downloaded from the Pfam database (Finn et al., 2015). The gene obtained from the first HMM search was aligned using CLUSTAL Omega (Sievers et al., 2011), and then the second HMM search was performed against the predicted pistachio proteins at E-value 0.01. The retrieved NBS genes were further searched for TIR and LRR motifs using HMM search and CC motifs were searched using Paircoil2 (P score of 0.025). NCBI CDD and Multiple Expectation Maximization for Motif Elicitation (MEME) were used to confirm the NBS, TIR, CC, and LRR domains (Marchler-Bauer et al., 2015). The distributions of the NBS-encoding genes across 15 chromosomes of cvs. Siirt and Bagyolu were analyzed.

**Analysis of Alternative Splicing (AS) Genes and Prediction of Protein Subcellular Locations**

A total of 126,707 unique transcripts with 1,452 contigs and 125,255 singlets were assembled using CAP3 following a previously described protocol (Min et al., 2015). Alternative splicing was analyzed by mapping the assembled unique transcripts to the corresponding genomic sequences of both male and female pistachio genomes (Min et al., 2015). The subcellular locations of proteins in both male and female plants were predicted using a well-developed protocol involving multiple software tools, including SignalP 4.0, TargetP, Phobius, WoLF PSORT, TMHMM, and PS-Scan (Lum and Min, 2013).

**SUPPLEMENTARY FIGURES**


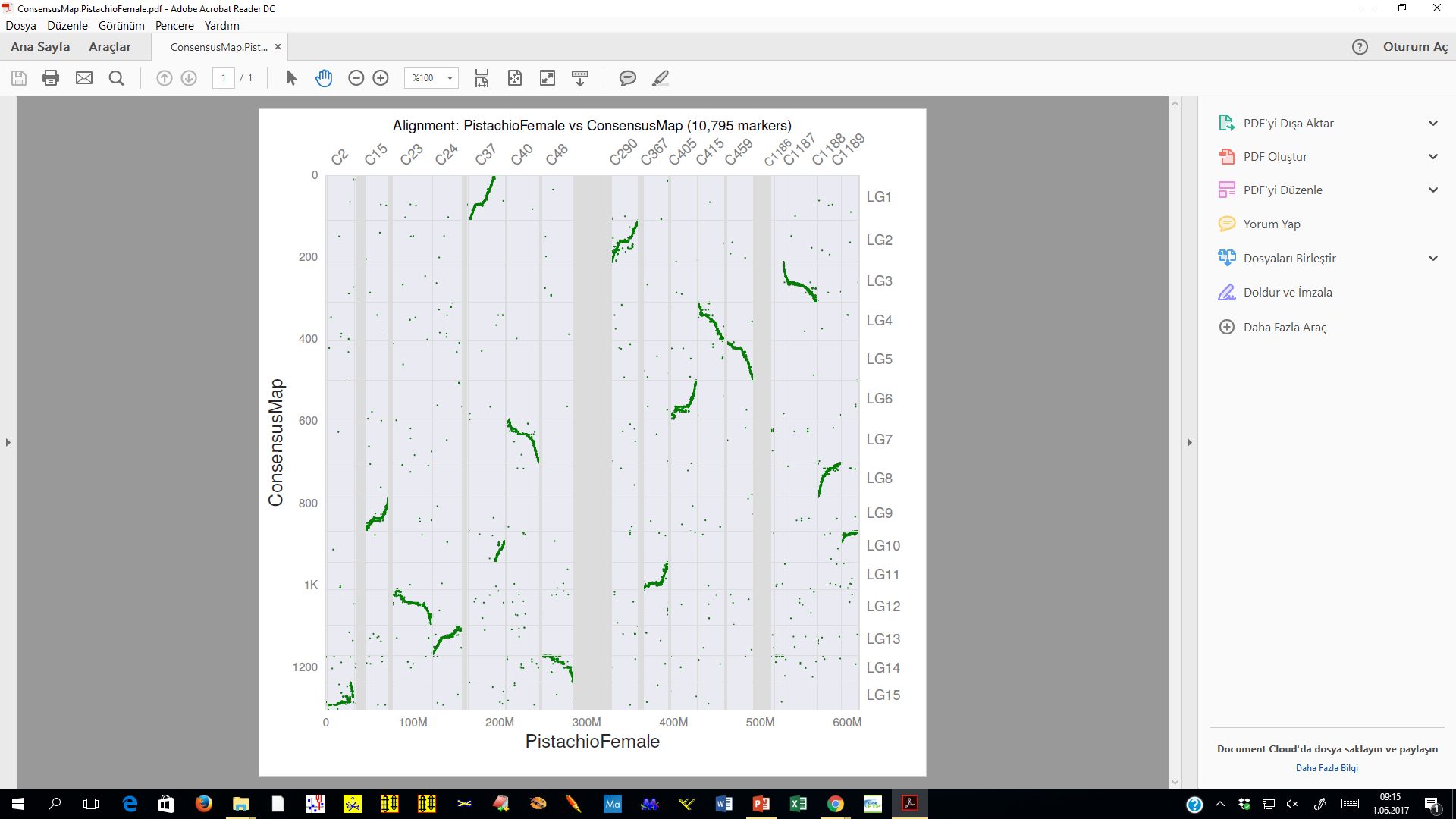


**Supplemental Figure 1.** Alignment of the Hi-C assembly and consensus genetic linkage map for cv. Siirt.

**
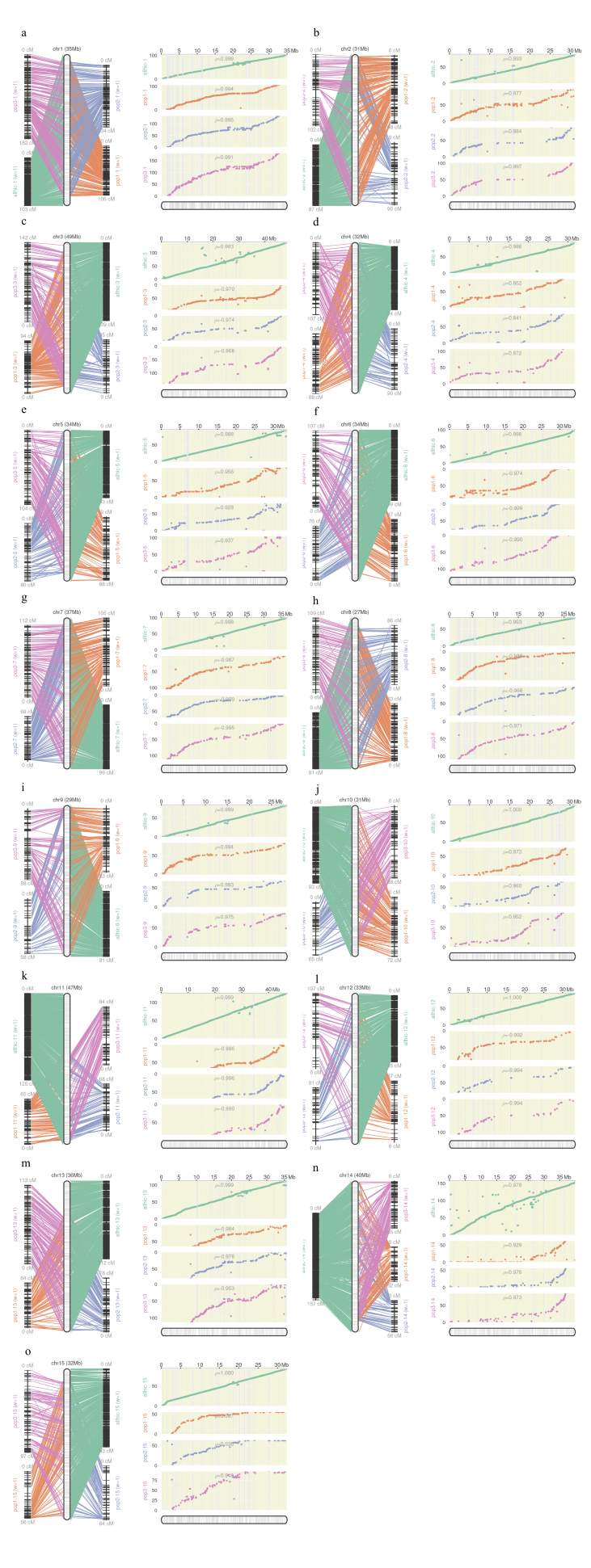
**

**Supplemental Figure 2.** Integration of the Hi-C assembly and consensus genetic linkage map for cv. Bagyolu (from a to o represents chromosomes 1 to 15, respectively).

**
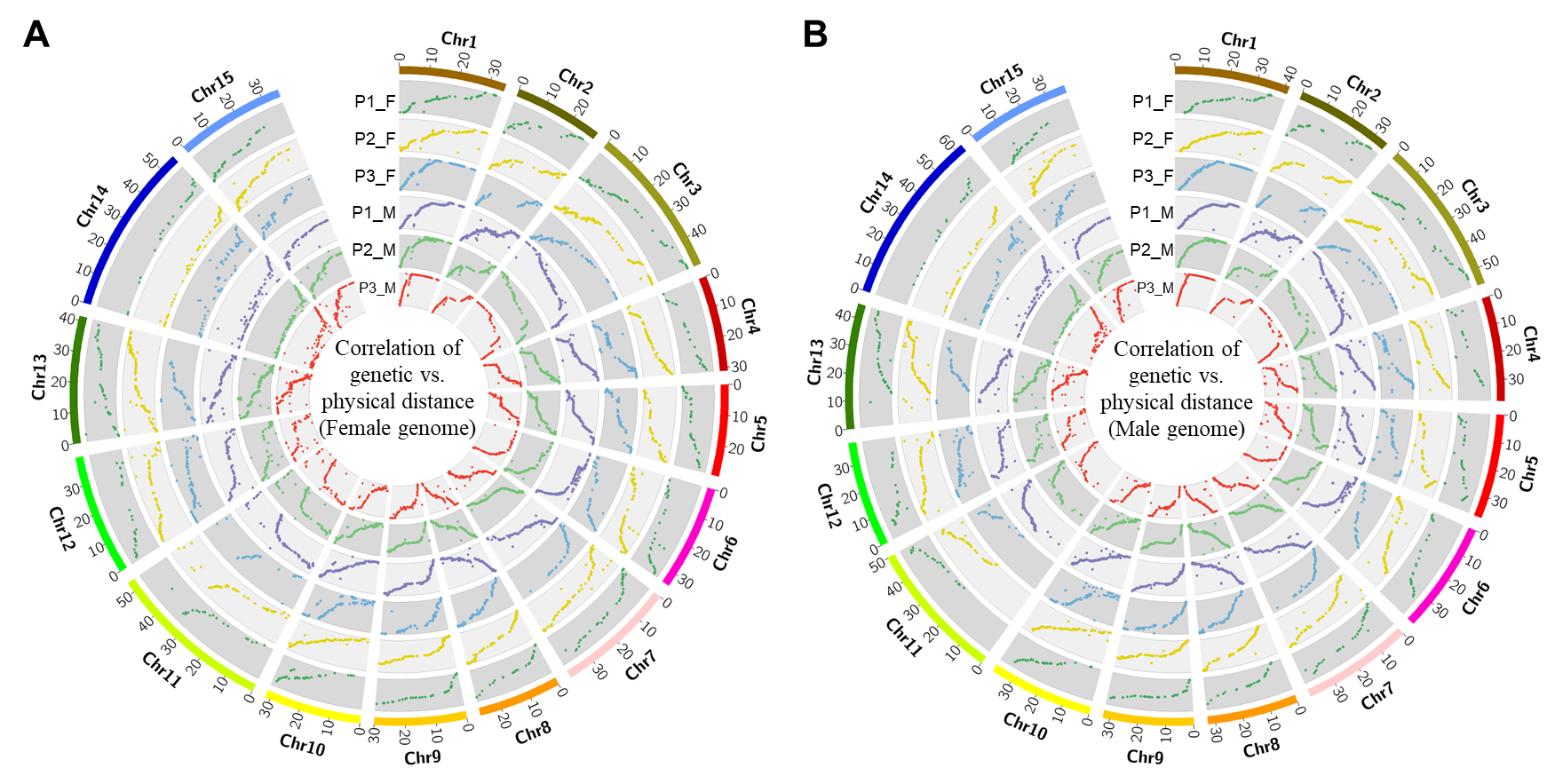
Supplemental Figure 3.** Circos plots showing the low correlation between genetic versus physical distcane of **chromosome 14** (sex chromosome) on the pistachio female Siirt genome (**A**) and male Bagyolu genome (**B**). The low-recombining sex chromosome were identified by scatter plot between genetic (cM) versus physical (Mb) positions showing clear and concordant curve reduction along **chromosome 14**. The outer ring to the inner ring represent the six genetic maps (Pop1_Female, Pop2_Female, Pop3_Female, Pop1_Male, Pop2_Male, Pop3_Male).

**
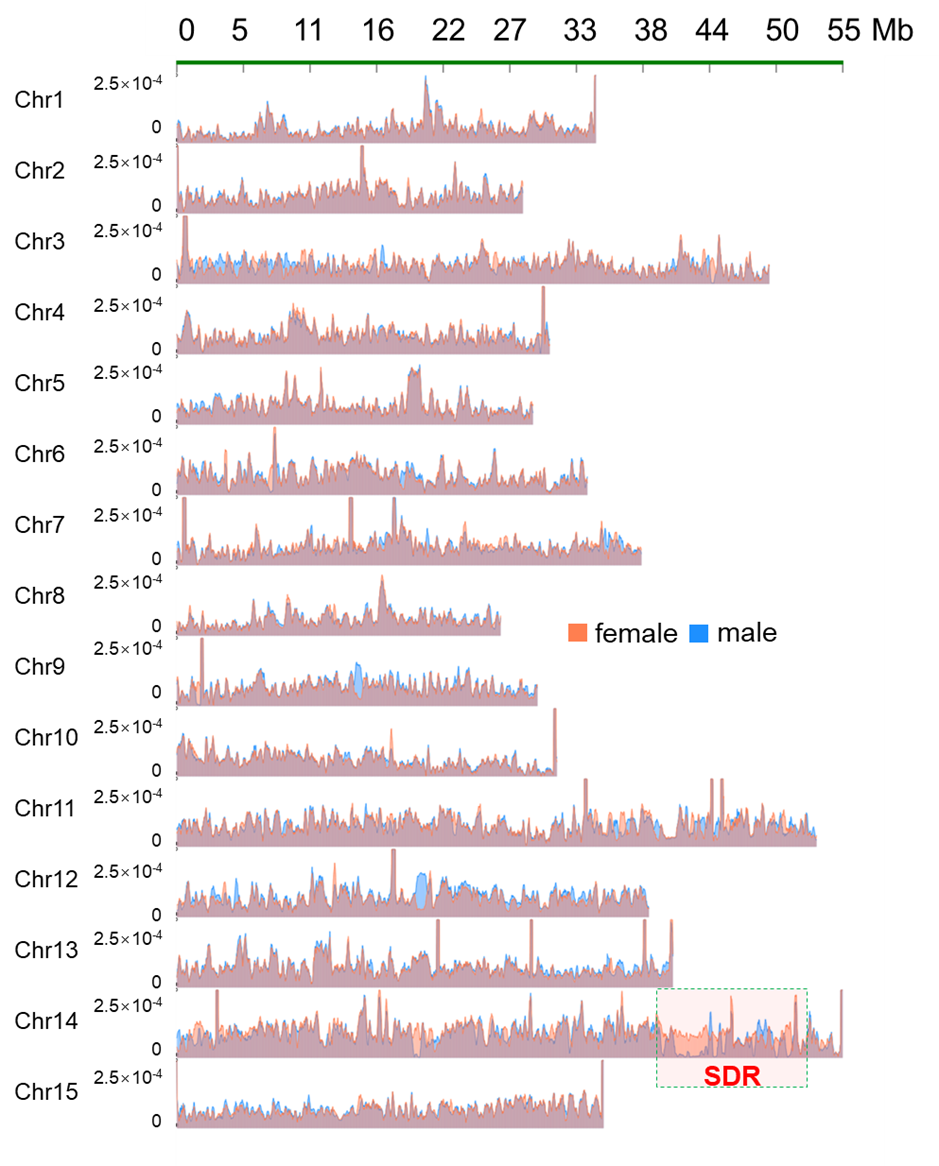
 Supplemental Figure 4.** Reads coverages between pooled resequenced females (nine individuals) and males (nine individuals) along the female pistachio Siirt genome.

**
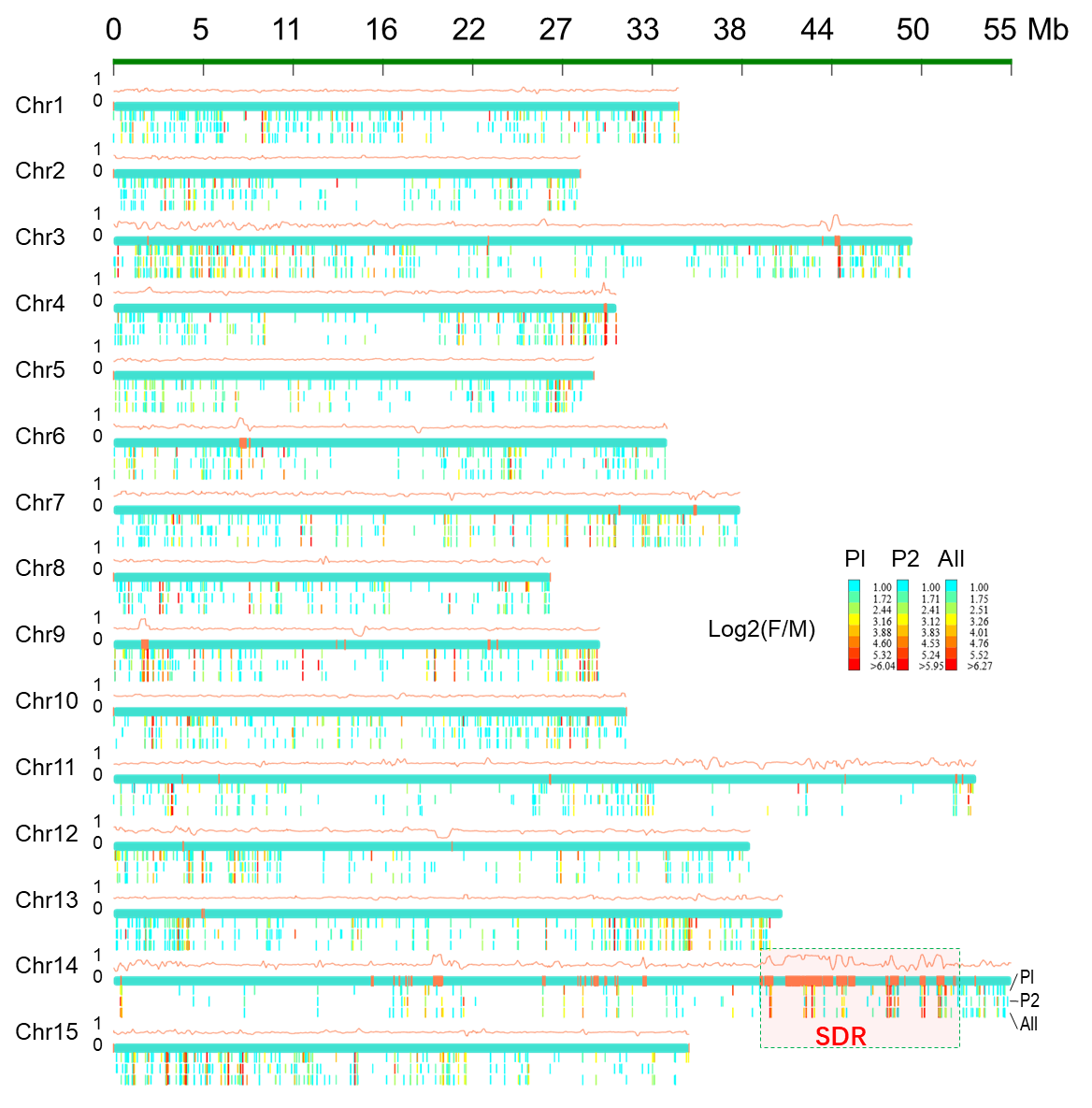
Supplemental Figure 5.** From top to bottom: 1) F/(F+M) ratios of reads coverages between pooled resequenced females (nine individuals) and males (nine individuals) along the female pistachio Siirt genome; 2) Distribution of female-specific SNPs; 3) The heatmap of DEGs (differentially expressed genes, showing log2F/M > 0) between females and males (log2F/M) at developmental period 1 (P1), period 2 (P2), and throughout all periods (All).

**
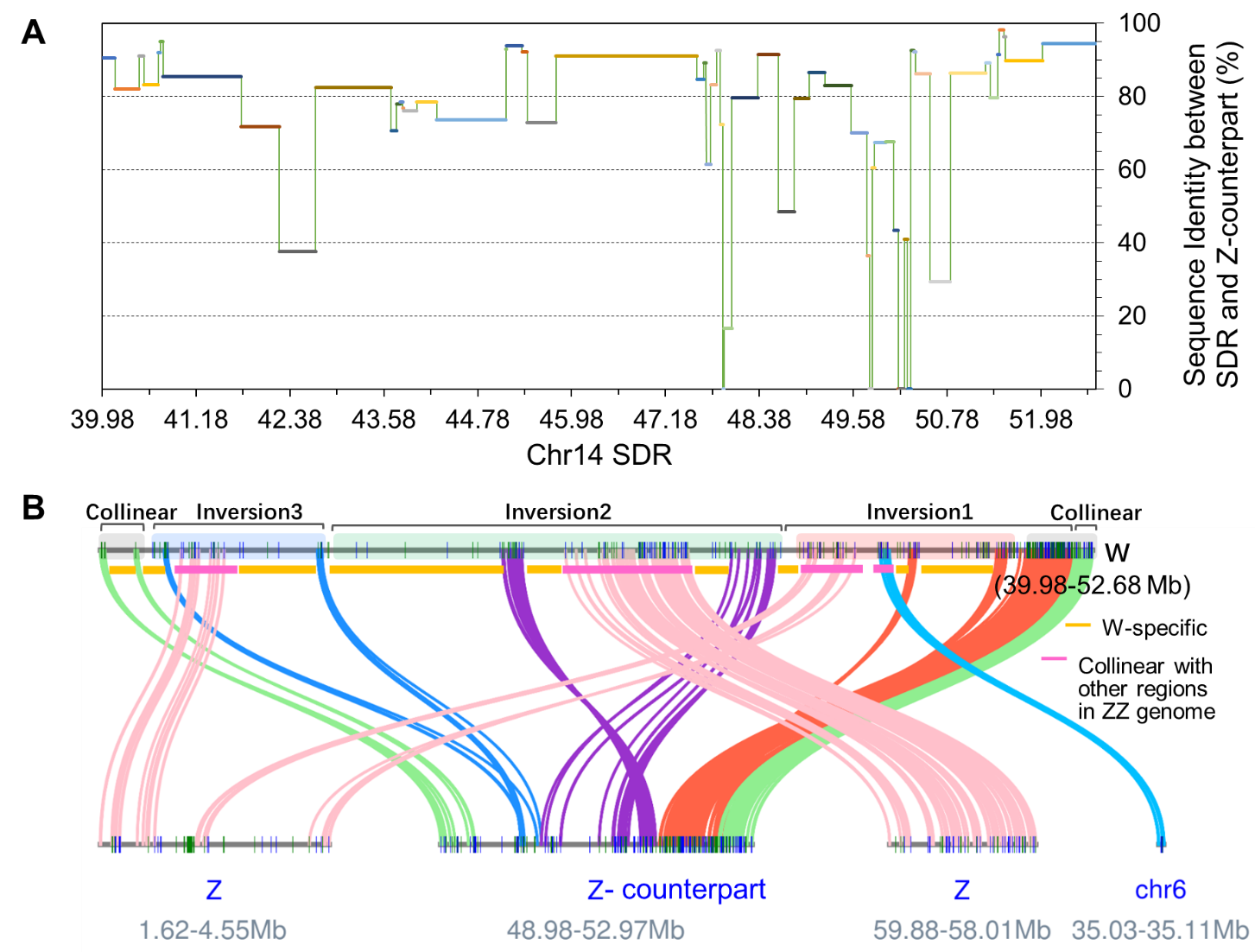
Supplemental Figure 6.** Sequencing identity between SDR and Z-counterpart as well as other collinear regions except Z-counterpart. (**A**) Plot of avergae sequence identity (%) of each contigs of SDR and the corresponding regions of Z-counterpart. Sliding window (1-kb window) approach were used to calculate the avergae sequence identity (%) for each contigs in SDR with the corresponding regions of Z-counterpart. The average sequence identity between SDR and Z-counterpart is 70.64%. Three small contigs (chr14:40723001-40758658; chr14:51450189-51506788; chr14:51506889-51528470) with average sequence identity >95% are located in collinear regions, while only one contig (chr14:51450189-51506788) with average sequence identity >98%. (**B**) Collinear genomic landscape of SDR (39.98-52.68 Mb) and ZZ genome inlcuding collinear Z-counterpart (48.98-52.97 Mb) and Collinear ZZ Chr14 (1.62-4.55 Mb, 59.88-58.01 Mb), as well as ZZ Chr6 (35.03-35.11 Mb).

**
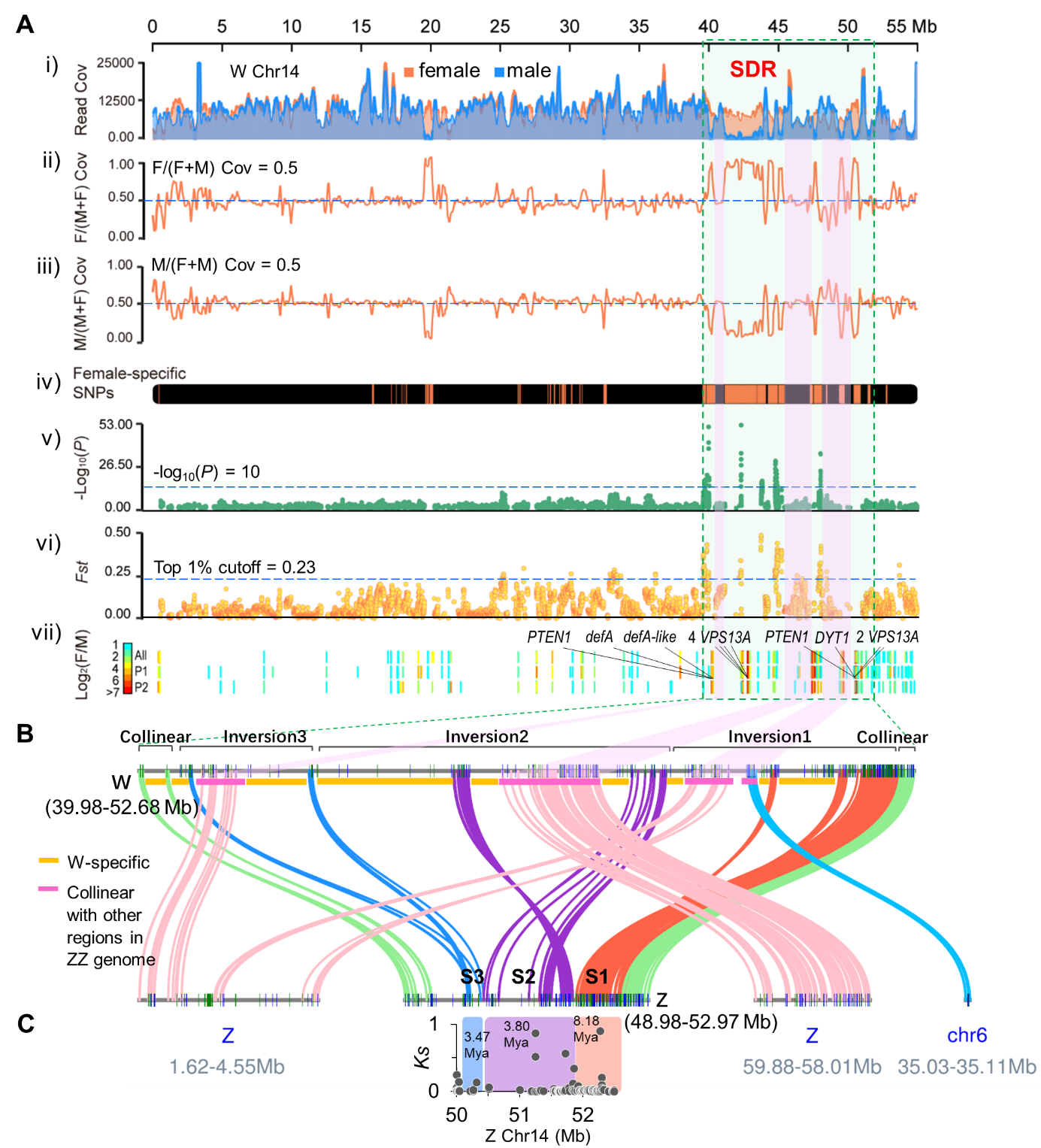
**

**Supplemental Figure 7.** Genomic features of SDR and Z-counterpart. (**A**) Evidence and boundaries of sex-determination regions (SDR) along Chr14 of female pistachio Siirt genome. From top to bottom of figure: i) read coverages between pooled resequenced females (9 individuals) and males (9 individuals); ii) the ratios F/(F+M) of females and males read coverages, with cutoff threshold of 0.5; iii) the ratios M/(F+M) of males and females read coverages, with cutoff threshold of 0.5; iv) female-specific SNPs showing continous blocks; v) Genome-wide association study (GWAS) plot between two sexual phenotypes, with cutoff of −log 10 (*P*) = 10; vi) *Fst* between females and males, with the top 1% cutoff peak; vii) the DEGs (differentially expressed genes, showing log2F/M > 0) between females and males in two different developmetal periods (P1, P2) and throughout all periods (All). Highligted genes are W-spcific SDR genes including *defA* (pistachio.v30109300), *defA-like* (pistachio.v30109290), *DYT1* (pistachio.v30112090), two *PTEN1* (pistachio.v30112050A, pistachio.v30109260) as well as two tandem duplications of *VPS13A* (two paralogs: pistachio.v30112100, v30112110; and four paralogs: pistachio.v30109730, v30109740, v30109750, v30109760). (**B**) Collinear genomic landscape of SDR (39.98-52.68 Mb) and ZZ genome inlcuding collinear Z-counterpart (48.98-52.97 Mb) and Collinear ZZ Chr14 (1.62-4.55 Mb), ZZ Chr14 (59.88-58.01 Mb) and ZZ Chr6 (35.03-35.11Mb). Three large chromosomal inversions on W Chr14: 40647874-52416681 (Size = 11.77 Mb), and corresponding Z Chr14: 50002496-52514087 (Size = 2.51 Mb) were indicated. W-specific sequences are marked with yellow blocks, regions collinear with other regions except Z-conterpart regions of ZZ genome are marked with pink blocks including Chr14: 41009767 - 41566853 (0.56 Mb), Chr14: 45933462 - 47505147 (1.57 Mb), Chr14: 49048829 - 49640049 (0.59 Mb), Chr14: 49958154 - 50081910 (0.12 Mb). (**C**) Plots of synonymous site divergence (*Ks*) of paired genes on the physical map of gene order along the Z chromosome. Inversions and collinear regions of the pistachio sex chromosomes Z and W chromosomes with different *Ks* and divergence times (Mya) are shown. The plots showing three evolutionary strata: stratum1, 2 and 3 (S1, 8.18 Mya; S2, 3.80 Mya; S3, 3.47 Mya).


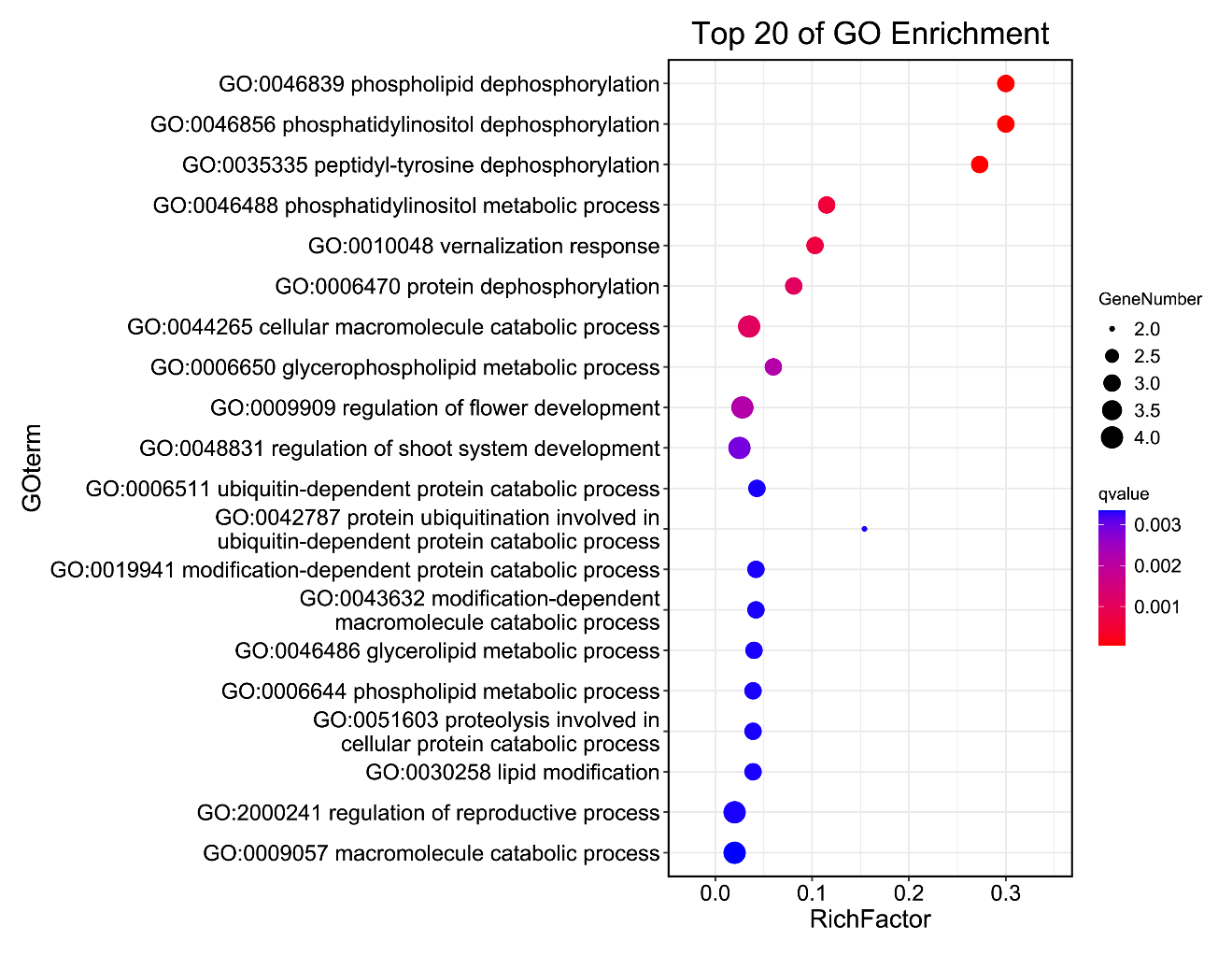


**Supplemental Figure 8A.** Top 20 significant (*P <*0.05, Fisher’s exact test) GOs enriched for the W-specific genes in Biological Process.


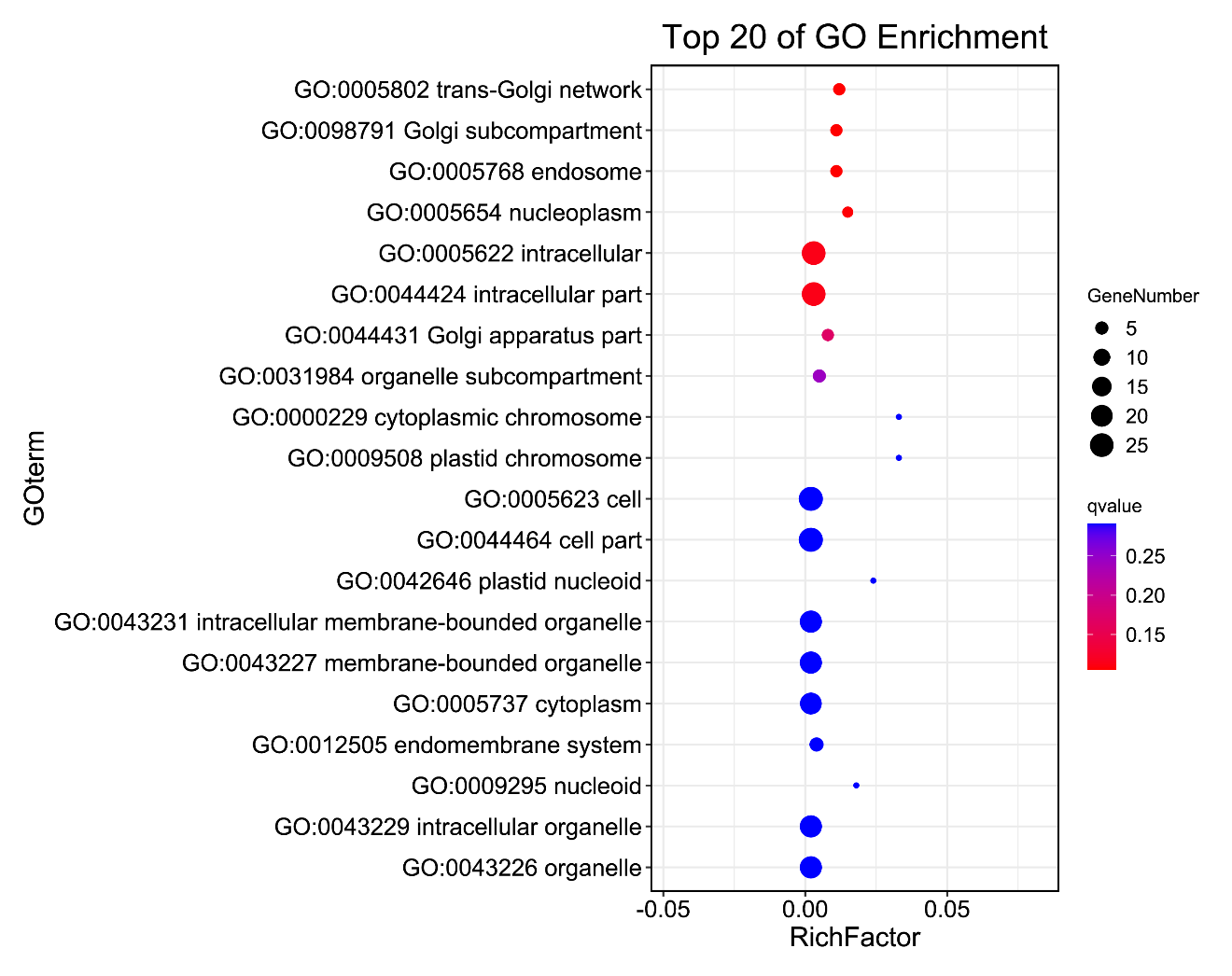


**Supplemental Figure 8B.** Top 20 significant (*P <*0.05, Fisher’s exact test) GOs enriched for the W-specific genes in Cellular Compenents.


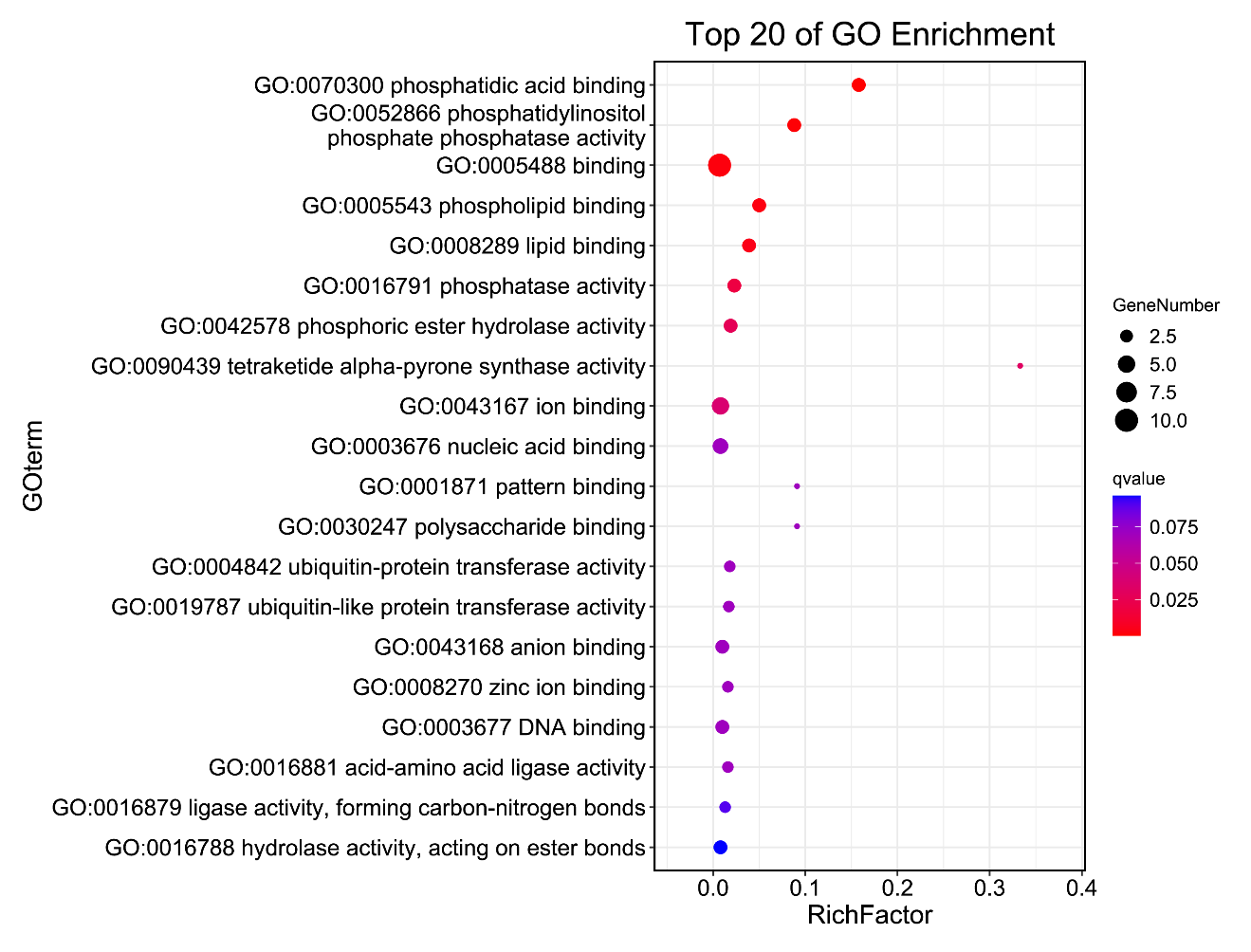


**Supplemental Figure 8C.** Top 20 significant (*P <*0.05, Fisher’s exact test) GOs enriched for the W-specific genes in Molecular Function.


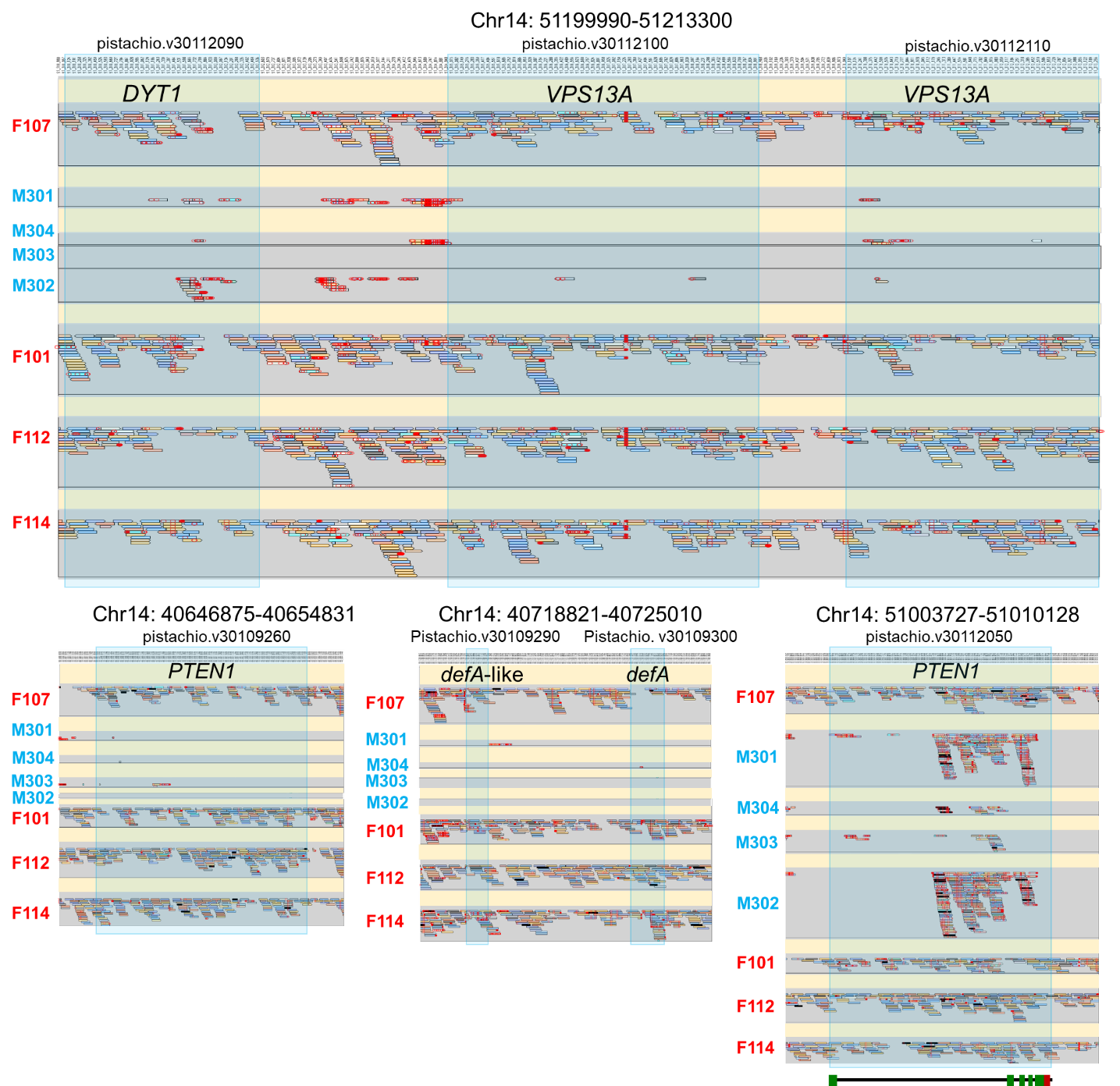


**Supplemental Figure 9.** Mapping reads of example accessions show the candidiate W-specific genes: Transcription factor *DYT1* (pistachio.v30112090); One tandem repeat with two *VPS13A* genes (pistachio.v30112100, v30112110); Two genes encoding pollen-specific phosphatase *PTEN1* (pistachio.v30112050, pistachio.v30109260); as well as one gene encoding a floral homeotic *defA* (pistachio.v30109300) and one gene encoding a floral homeotic *defA*-like protein (pistachio.v30109290) presenting in resequencing females and absent in male accessions.


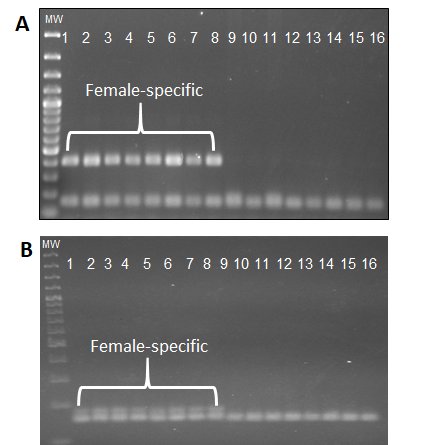


**Supplemental Figure 10.** Image of agarose gel electrophoresis of PCR-amplified (**A**) pistachio.v30109290 and (**B**) pistachio.v30109300 genes from pistachio cultivars and accessions. (Female cultivars: 1. Siirt, 2.Ohadi, 3. Kalehghouchi, 4. Ashoury, 5. Kerman, 6. Uzun, 7. Beyaz Ben, 8. Bilgen; and Male cultivars: 9. Kaska, 10. Bagyolu, 11. Atli, 12. Peters, 13. Uygur, 14. Male3, 15. Male7, 16. M23). The bands with lowest molecular weight in each gel is a positive control (pistachio.v30134120).


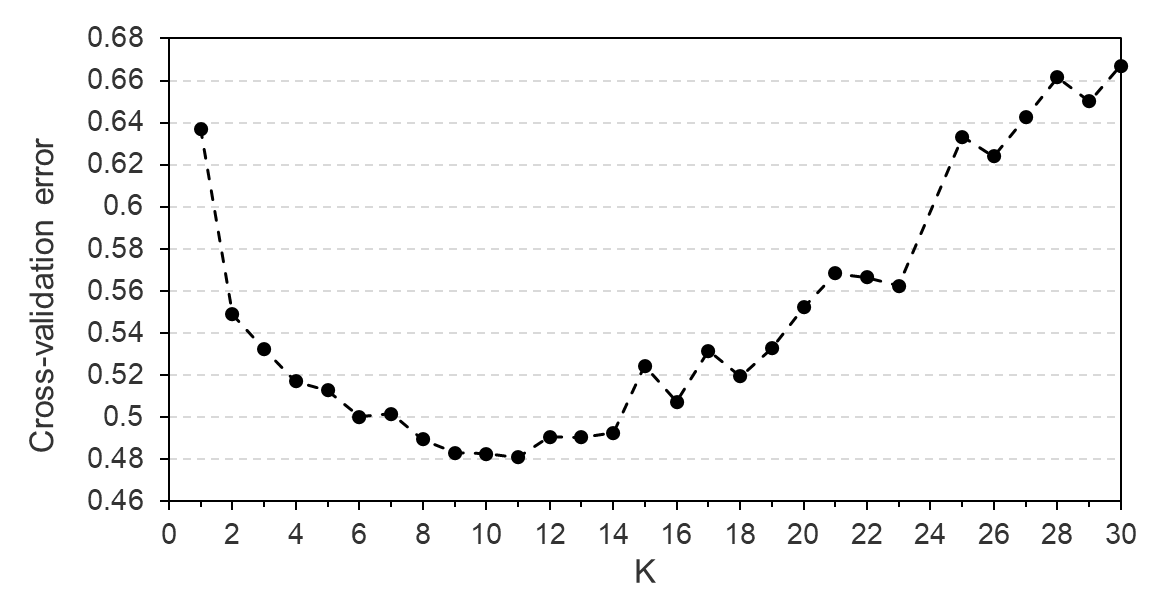


**Supplemental Figure 11.** Cross-validation error analysis shows the optimal population stratification of population structure is K = 11.

**
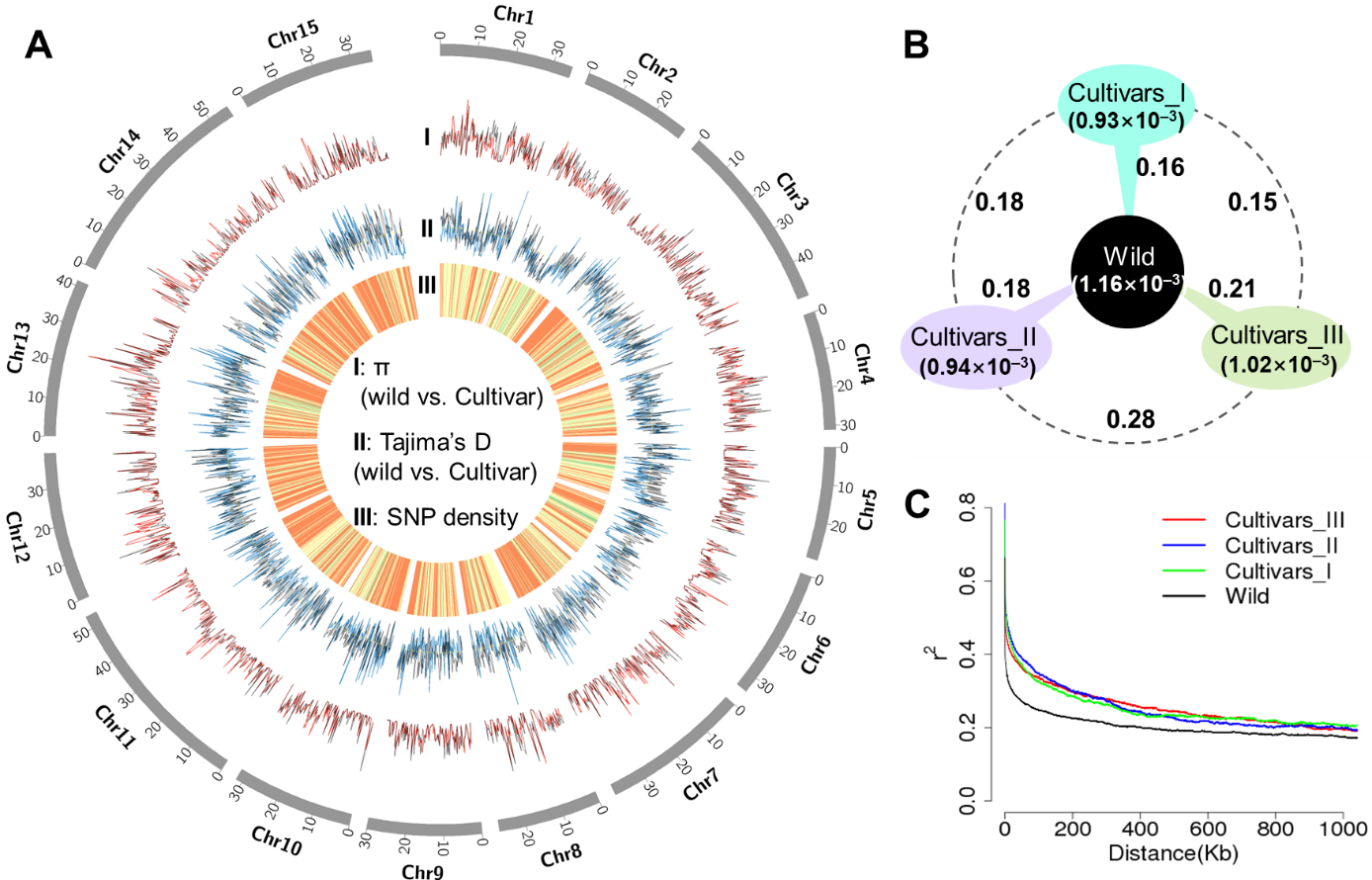
**

**Supplemental Figure 12.** Genome-wide genetic diversity of 160 domesticated *P. vera* accessions compared to 14 wild *P. vera* accessions. (**A**) Nucleotide diversity, Tajima’s D and SNP density are shown from the outer to the center rings of the Circos plot. In ring A, the red line represents cultivars, the black line represents wild population; in ring B, the blue line represents cultivars, the black line represents wild population; ring C indicates the SNP density of all the accessions. (**B**) Summary of nucleotide diversity and population divergence across the wild group and Cultivar_I, Cultivar_II, and Cultivar_III groups. Values in parentheses represent measures of nucleotide diversity for the group, and values between pairs indicate population divergence (*F*st). (c) LD decay for the three cultivated sub-groups and the wild sub-group.

**SUPPLEMENTARY REFERENCES**

**Bao, W., Kojima, K. K., and Kohany, O.** (2015). Repbase Update, a database of repetitive elements in eukaryotic genomes. Mob. DNA **6**:1-6. https://doi:10.1186/s13100-015-0041-9.

**Benson, G.** (1999). Tandem repeats finder: A program to analyze DNA sequences. Nucleic Acids Res. **27**:573–580. https://doi.org/10.1093/nar/27.2.573.

**Berlin, K., Koren, S., Chin, C. S., Drake, J. P., Landolin, J. M., and Phillippy, A. M.** (2015). Assembling large genomes with single-molecule sequencing and locality-sensitive hashing. Nat. Biotechnol. **33**:623. https://doi.org/10.1038/nbt.3238.

**Boetzer, M., Henkel, C. V., Jansen, H. J., Butler, D., and Pirovano, W.** (2011). Scaffolding pre-assembled contigs using SSPACE. Bioinformatics **27**:578. https://doi.org/10.1093/bioinformatics/btq683.

**Cantarel, B. L., Korf, I., Robb, S. M. C., Parra, G., Ross, E., Moore, B., Holt, C., Alvarado, A. S., and Yandell, M.** (2008). MAKER: An easy-to-use annotation pipeline designed for emerging model organism genomes. Genome Res. **18**:188. http:// doi/10.1101/gr.6743907.

**Depristo, M. A., Banks, E., Poplin, R., Garimella, K. V., Maguire, J. R., Hartl, C., Philippakis, A. A., Del Angel, G., Rivas, M. A., Hanna, M., et al.** (2011). A framework for variation discovery and genotyping using next-generation DNA sequencing data. Nat. Genet. **43**:491-498. https://doi:10.1038/ng.806.

**Finn, R. D., Clements, J., Arndt, W., Miller, B. L., Wheeler, T. J., Schreiber, F., Bateman, A., and Eddy, S. R.** (2015). HMMER web server: 2015 Update. Nucleic Acids Res. **43**:W30-W38. https://doi:10.1093/nar/gkv397.

**Gnerre, S., MacCallum, I., Przybylski, D., Ribeiro, F. J., Burton, J. N., Walker, B. J., Sharpe, T., Hall, G., Shea, T. P., Sykes, S., et al.** (2011). High-quality draft assemblies of mammalian genomes from massively parallel sequence data. Proc. Natl. Acad. Sci. U. S. A. **108**:1513-1518. https://doi:10.1073/pnas.1017351108.

**Grabherr, M. G., Haas, B. J., Yassour, M., Levin, J. Z., Thompson, D. A., Amit, I., Adiconis, X., Fan, L., Raychowdhury, R., Zeng, Q., et al.** (2011). Full-length transcriptome assembly from RNA-Seq data without a reference genome. Nat. Biotechnol. **29**:644. https://doi.org/10.1038/nbt.1883.

**Haas, B. J., Salzberg, S. L., Zhu, W., Pertea, M., Allen, J. E., Orvis, J., White, O., Robin, C. R., and Wortman, J. R.** (2008). Automated eukaryotic gene structure annotation using EVidenceModeler and the Program to Assemble Spliced Alignments. Genome Biol. **9**:R7. https://doi.org/10.1186/gb-2008-9-1-r7.

**Huang, X., and Madan, A.** (1999). CAP3: A DNA sequence assembly program. Genome Res. **9**:868–877. https://doi.org/10.1101/gr.9.9.868.

**Huerta-Cepas, J., Forslund, K., Coelho, L. P., Szklarczyk, D., Jensen, L. J., Von Mering, C., and Bork, P.** (2017). Fast genome-wide functional annotation through orthology assignment by eggNOG-mapper. Mol. Biol. Evol. **34**:2115-2122. https://doi:10.1093/molbev/msx148.

**Kalvari, I., Argasinska, J., Quinones-Olvera, N., Nawrocki, E. P., Rivas, E., Eddy, S. R., Bateman, A., Finn, R. D., and Petrov, A. I.** (2018). Rfam 13.0: Shifting to a genome-centric resource for non-coding RNA families. Nucleic Acids Res. **46**:335–342. https://doi.org/10.1093/nar/gkx1038.

**Koren, S., Walenz, B. P., Berlin, K., Miller, J. R., Bergman, N. H., and Phillippy, A. M.** (2017). Canu: Scalable and accurate long-read assembly via adaptive κ-mer weighting and repeat separation. Genome Res. **27**:722-736. https://doi:10.1101/gr.215087.116.

**Korf, I.** (2004). Gene finding in novel genomes. BMC Bioinformatics **5**:59. https://doi.org/10.1186/1471-2105-5-59.

**Kozomara, A., and Griffiths-Jones, S.** (2014). MiRBase: Annotating high confidence microRNAs using deep sequencing data. Nucleic Acids Res. **42**:D68–D73. https://doi.org/10.1093/nar/gkt1181.

**Langmead, B., Trapnell, C., Pop, M., and Salzberg, S. L.** (2009). Ultrafast and memory-efficient alignment of short DNA sequences to the human genome. Genome Biol. **10**:1-10. https://doi:10.1186/gb-2009-10-3-r25.

**Li, H., and Durbin, R.** (2010). Fast and accurate long-read alignment with Burrows-Wheeler transform. Bioinformatics **25**:1754. https://doi.org/10.1093/bioinformatics/btp698.

**Lieberman-Aiden, E., Van Berkum, N. L., Williams, L., Imakaev, M., Ragoczy, T., Telling, A., Amit, I., Lajoie, B. R., Sabo, P. J., Dorschner, M. O., et al.** (2009). Comprehensive mapping of long-range interactions reveals folding principles of the human genome. Science . **326**:289–293. https://doi.org/10.1126/science.1181369.

**Lomsadze, A., Ter-Hovhannisyan, V., Chernoff, Y. O., and Borodovsky, M.** (2005). Gene identification in novel eukaryotic genomes by self-training algorithm. Nucleic Acids Res. **33**:6494. https://doi.org/10.1093/nar/gki937.

**Lowe, T. M., and Chan, P. P.** (2016). tRNAscan-SE On-line: integrating search and context for analysis of transfer RNA genes. Nucleic Acids Res. **44**:54–57. https://doi.org/10.1093/nar/gkw413.

**Lowe, T. M., and Eddy, S. R.** (1997). tRNAscan-SE: A Program for Improved Detection of Transfer RNA Genes in Genomic Sequence. Nucleic Acids Res. **25**:955-964. https://doi:10.1093/nar/25.5.955.

**Lum, G., and Min, X. J.** (2013). Bioinformatic protocols and the knowledge-base for secretomes in fungi. In *Laboratory Protocols in Fungal Biology: Current Methods in Fungal Biology* (ed. Gupta, V.K., Tuohy, M.G., Ayyachamy, M., Turner, K.M., O’Donovan, A.), pp. 545–557. New York: Springer.

**Luo, R., Liu, B., Xie, Y., Li, Z., Huang, W., Yuan, J., He, G., Chen, Y., Pan, Q., Liu, Y., et al.** (2012). SOAPdenovo2: An empirically improved memory-efficient short-read de novo assembler. Gigascience **1**:2047-217X. https://doi:10.1186/2047-217X-1-18.

**Marchler-Bauer, A., Derbyshire, M. K., Gonzales, N. R., Lu, S., Chitsaz, F., Geer, L. Y., Geer, R. C., He, J., Gwadz, M., Hurwitz, D. I., et al.** (2015). CDD: NCBI’s conserved domain database. Nucleic Acids Res. **43**:D222-D226. https://doi:10.1093/nar/gku1221.

**Meyers, B. C., Axtell, M. J., Bartel, B., Bartel, D. P., Baulcombe, D., Bowman, J. L., Cao, X., Carrington, J. C., Chen, X., Green, P. J., et al.** (2008). Criteria for annotation of plant microRNAs. Plant Cell **20**:3186-3190. http://doi:10.1105/tpc.108.064311.

**Min, X. J., Powell, B., Braessler, J., Meinken, J., Yu, F., and Sablok, G.** (2015). Genome-wide cataloging and analysis of alternatively spliced genes in cereal crops. BMC Genomics **16**:721. https://doi.org/10.1186/s12864-015-1914-5.

**Morgulis, A., Gertz, E. M., Schäffer, A. A., and Agarwala, R.** (2006). WindowMasker: Window-based masker for sequenced genomes. Bioinformatics **22**:134-141. https://doi:10.1093/bioinformatics/bti774.

**Nawrocki, E. P.** (2014). Annotating functional RNAs in genomes using infernal. Methods Mol. Biol. **1097**:163–197. https://doi.org/10.1007/978-1-62703-709-9_9.

**Nawrocki, E. P., and Eddy, S. R.** (2013). Infernal 1.1: 100-fold faster RNA homology searches. Bioinformatics **29**:2933–2935. https://doi.org/10.1093/bioinformatics/btt509.

**Novák, P., Neumann, P., and Macas, J.** (2010). Graph-based clustering and characterization of repetitive sequences in next-generation sequencing data. BMC Bioinformatics **11**:378. https://doi.org/10.1186/1471-2105-11-378.

**Parra, G., Bradnam, K., and Korf, I.** (2007). CEGMA: a pipeline to accurately annotate core genes in eukaryotic genomes. Bioinformatics **23**:1061–1067. https://doi.org/10.1093/bioinformatics/btm071.

**Pryszcz, L. P., and Gabaldón, T.** (2016). Redundans: An assembly pipeline for highly heterozygous genomes. Nucleic Acids Res. **44**:e113. https://doi.org/10.1093/nar/gkw294.

**Putnam, N. H., O’Connell, B. L., Stites, J. C., Rice, B. J., Blanchette, M., Calef, R., Troll, C. J., Fields, A., Hartley, P. D., Sugnet, C. W., et al.** (2016). Chromosome-scale shotgun assembly using an in vitro method for long-range linkage. Genome Res. **26**: 342-350. https://doi:10.1101/gr.193474.115.

**Sakai, H., Naito, K., Ogiso-Tanaka, E., Takahashi, Y., Iseki, K., Muto, C., Satou, K., Teruya, K., Shiroma, A., Shimoji, M., et al.** (2015). The power of single molecule real-time sequencing technology in the de novo assembly of a eukaryotic genome. Sci. Rep. **5**:16780. https://doi:10.1038/srep16780 (2015).

**Sievers, F., Wilm, A., Dineen, D., Gibson, T. J., Karplus, K., Li, W., Lopez, R., McWilliam, H., Remmert, M., Söding, J., et al.** (2011). Fast, scalable generation of high-quality protein multiple sequence alignments using Clustal Omega. Mol. Syst. Biol. **7**:539. https://doi:10.1038/msb.2011.75.

**Simão, F. A., Waterhouse, R. M., Ioannidis, P., Kriventseva, E. V., and Zdobnov, E. M.** (2015). BUSCO: Assessing genome assembly and annotation completeness with single-copy orthologs. Bioinformatics **31**:3210–3212. https://doi.org/10.1093/bioinformatics/btv351.

**Stanke, M., Schöffmann, O., Morgenstern, B., and Waack, S.** (2006). Gene prediction in eukaryotes with a generalized hidden Markov model that uses hints from external sources. BMC Bioinformatics **7**:62. https://doi.org/10.1186/1471-2105-7-62.

**Tang, H., Zhang, X., Miao, C., Zhang, J., Ming, R., Schnable, J. C., Schnable, P. S., Lyons, E., and Lu, J.** (2015). ALLMAPS: Robust scaffold ordering based on multiple maps. Genome Biol. **16**:3. https://doi.org/10.1186/s13059-014-0573-1.

**Thompson, J. D., Gibson, T. J., Plewniak, F., Jeanmougin, F., and Higgins, D. G.** (1997). The CLUSTAL X windows interface: Flexible strategies for multiple sequence alignment aided by quality analysis tools. Nucleic Acids Res. **24**:4876–4882. https://doi.org/10.1093/nar/25.24.4876.

**Walker, B. J., Abeel, T., Shea, T., Priest, M., and Abouelliel, A.** (2014). Pilon: An Integrated Tool for Comprehensive Microbial Variant Detection and Genome Assembly Improvement. PLoS One **9**:e112963. https://doi.org/10.1371/journal.pone.0112963.

**Zhang, Z., Yu, J., Li, D., Zhang, Z., Liu, F., Zhou, X., Wang, T., Ling, Y., and Su, Z.** (2009). PMRD: Plant microRNA database. Nucleic Acids Res. **38**:806–813. https://doi.org/10.1093/nar/gkp818.
